# Testing polymineral post‐IR IRSL and quartz SAR‐OSL protocols on Middle to Late Pleistocene loess at Batajnica, Serbia

**DOI:** 10.1111/bor.12442

**Published:** 2020-05-04

**Authors:** Anca Avram, Daniela Constantin, Daniel Veres, Szabolcs Kelemen, Igor Obreht, Ulrich Hambach, Slobodan B. Marković, Alida Timar‐Gabor

**Affiliations:** ^1^ Interdisciplinary Research Institute on Bio‐Nano‐Sciences Babes‐Bolyai University Treboniu Laurian 42 400271 Cluj‐Napoca Romania; ^2^ Faculty of Environmental Sciences and Engineering Babes‐Bolyai University Fantanele 30 400294 CLuj‐Napoca Romania; ^3^ Institute of Speleology Romanian Academy Clinicilor 5 400006 Cluj‐Napoca Romania; ^4^ Organic Geochemistry Group MARUM‐Center for Marine Environmental Sciences and Department of Geosciences University of Bremen Leobener 8 28359 Bremen Germany; ^5^ BayCEER& Chair of Geomorphology University of Bayreuth Universitätsstrasse 30 95440 Bayreuth Germany; ^6^ Chair of Physical Geography Faculty of Sciences University of Novi Sad Trg Dositeja Obradovica 3 21000 Novi Sad Serbia

## Abstract

The loess–palaeosol sequence of Batajnica (Vojvodina region, Serbia) is considered as one of the most complete and thickest terrestrial palaeoclimate archives for the Middle and Late Pleistocene. In order to achieve a numerical chronology for this profile, four sets of ages were obtained on 18 individual samples. Equivalent doses were determined using the SAR protocol on fine (4–11 μm) and coarse (63–90 μm) quartz fractions, as well as on polymineral fine grains by using two elevated temperature infrared stimulation methods, pIRIR_290_ and pIRIR_225_. We show that the upper age limit of coarse quartz OSL and polymineral pIRIR_290_ and pIRIR_225_ techniques is restricted to the Last Glacial/Interglacial cycle due to the field saturation of the natural signals. Luminescence ages on coarse quartz, pIRIR_225_ and pIRIR_290_ polymineral fine grains are in general agreement. Fine quartz ages are systematically lower than the coarse quartz and pIRIR ages, the degree of underestimation increasing with age. Comparison between natural and laboratory dose response curves indicate the age range over which each protocol provides reliable ages. For fine and coarse quartz, the natural and laboratory dose response curves overlap up to ~150 and ~250 Gy, respectively, suggesting that the SAR protocol provides reliable ages up to *c*. 50 ka on fine quartz and *c. *100 ka on coarse quartz. Using the pIRIR_225_ and pIRIR_290_ protocols, equivalent doses up to ~400 Gy can be determined, beyond which in the case of the former the natural dose response curve slightly overestimates the laboratory dose response curve. Our results suggest that the choice of the mineral and luminescence technique to be used for dating loess sediments should take into consideration the reported limited reliability.

The Middle Danube basin comprises several high‐resolution Middle to Late Pleistocene loess–palaeosol sequences (LPS) that document clear similarity in the long‐term palaeoclimate response between European and Asian loess deposits (Buggle *et al*. 2009, 2013; Marković *et al*. 2015 and references therein; Sümegi *et al*. 2018; Perić *et al*. 2019). This similarity allowed for inferring common variability in regional atmospheric circulation patterns and dust dynamics driven by glacial‐to‐interglacial climate variability (e.g. Marković *et al*. 2015; Zeeden *et al*. 2018a). However, reliable numerical age constraints for Middle Danube LPS are rare (Stevens *et al*. 2011; Thiel *et al*. 2011a; Murray *et al*. 2014; Bösken *et al*. 2017; Zhang *et al*. 2018; Perić *et al*. 2019). Therefore, the most common approach in deriving chronological frameworks for Middle Danube LPS has relied on correlative techniques based on variability in magnetic susceptibility data (e.g. Marković *et al*. 2006, 2015, 2018a; Basarin *et al*. 2014; Zeeden *et al*. 2018a). Magnetic susceptibility, a proxy for pedogenetic intensity in loess (Schaetzl *et al*. 2018; Zeeden *et al*. 2018b), allows the tentative alignment of loess records onto the benthic isotope stack (Lisiecki & Raymo 2005) or the insolation curve (Laskar *et al*. 2004).

Luminescence dating is the most applicable method in directly dating the emplacement time of loess‐forming mineral particles (Roberts 2008). To date, luminescence dating has been applied on quartz and feldspars extracted from Serbian LPS (Fuchs *et al*. 2008; Stevens *et al*. 2011; Timar‐Gabor *et al*. 2015; Perić *et al*. 2019) mainly for records covering the Last Glacial Cycle (LGC). Beyond the LGC, only Murray *et al*. (2014) have reported minimum pIRIR_290_ ages for samples found to be in field and laboratory saturation. They determined minimum ages derived from minimum equivalent dose estimates based on an upper limit of 86% of saturation of the laboratory dose response curves.

At the Batajnica LPS (Fig. 1) discussed here, five clearly distinguishable loess and palaeosol units are exposed, reaching to Marine Isotope Stage (MIS) 16 and possibly beyond (Marković *et al*. 2009). Despite the importance of the Batajnica LPS as a terrestrial palaeoclimate record for the Middle Danube loess field, no numerical ages have been reported so far.

**Figure 1:**
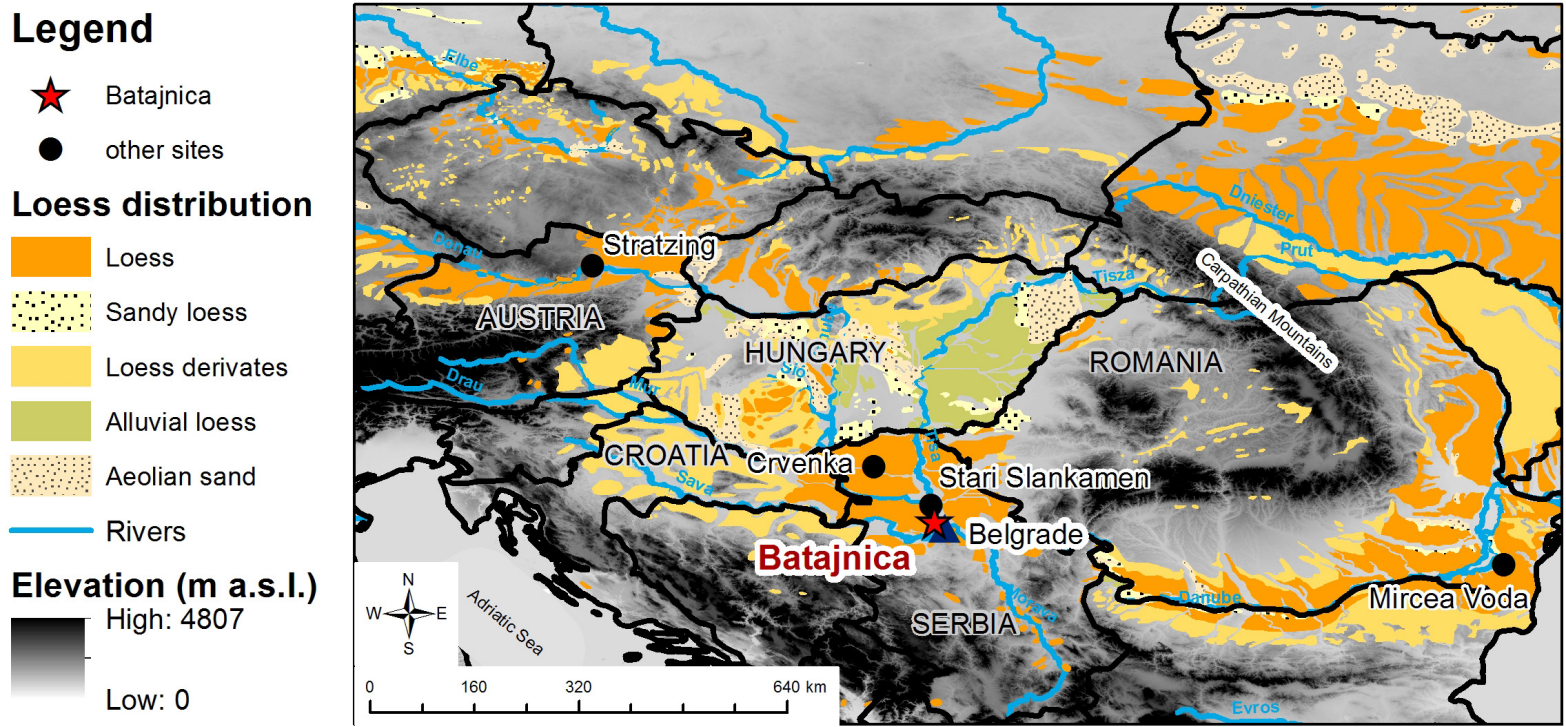
The study area integrated in the map of loess distribution after Haase *et al*. (2007). SRTM data provided by U.S. Geological Survey. The star represents the investigated loess site, while loess sites mentioned in this paper are marked by solid circles (Mircea‐Voda, Romania (Timar‐Gabor *et al*. 2011; Vasiliniuc *et al*. 2012); Stari Slankamen, Serbia (Murray *et al*. 2014); Crvenka, Serbia (Stevens *et al*. 2011); Stratzing, Austria (Thiel *et al*. 2011a)).

Here we (i) provide a detailed multi‐method luminescence chronology for the Batajnica profile with the focus on the last glacial loess unit; (ii) explore the upper dating limit of the single‐aliquot regenerative dose (SAR) protocol applied on 4–11 and 63–90 μm quartz OSL, as well as pIRIR_290_ and pIRIR_225_ applied on 4–11 μm polymineral fine grains from samples collected from L1 (*c*. MIS 5–2) loess unit and at the lower boundaries of S1 (*c*. MIS 5), S2 (*c*. MIS 7) and S3 (*c*. MIS 9) palaeosols; and (iii) compare the natural and laboratory generated quartz SAR‐OSL and post‐IR IRSL_225_ and IRSL_290_ dose response curves and thus assess what is the dose range over which each protocol provides reliable ages.

## Sampling and site description

**Figure 2:**
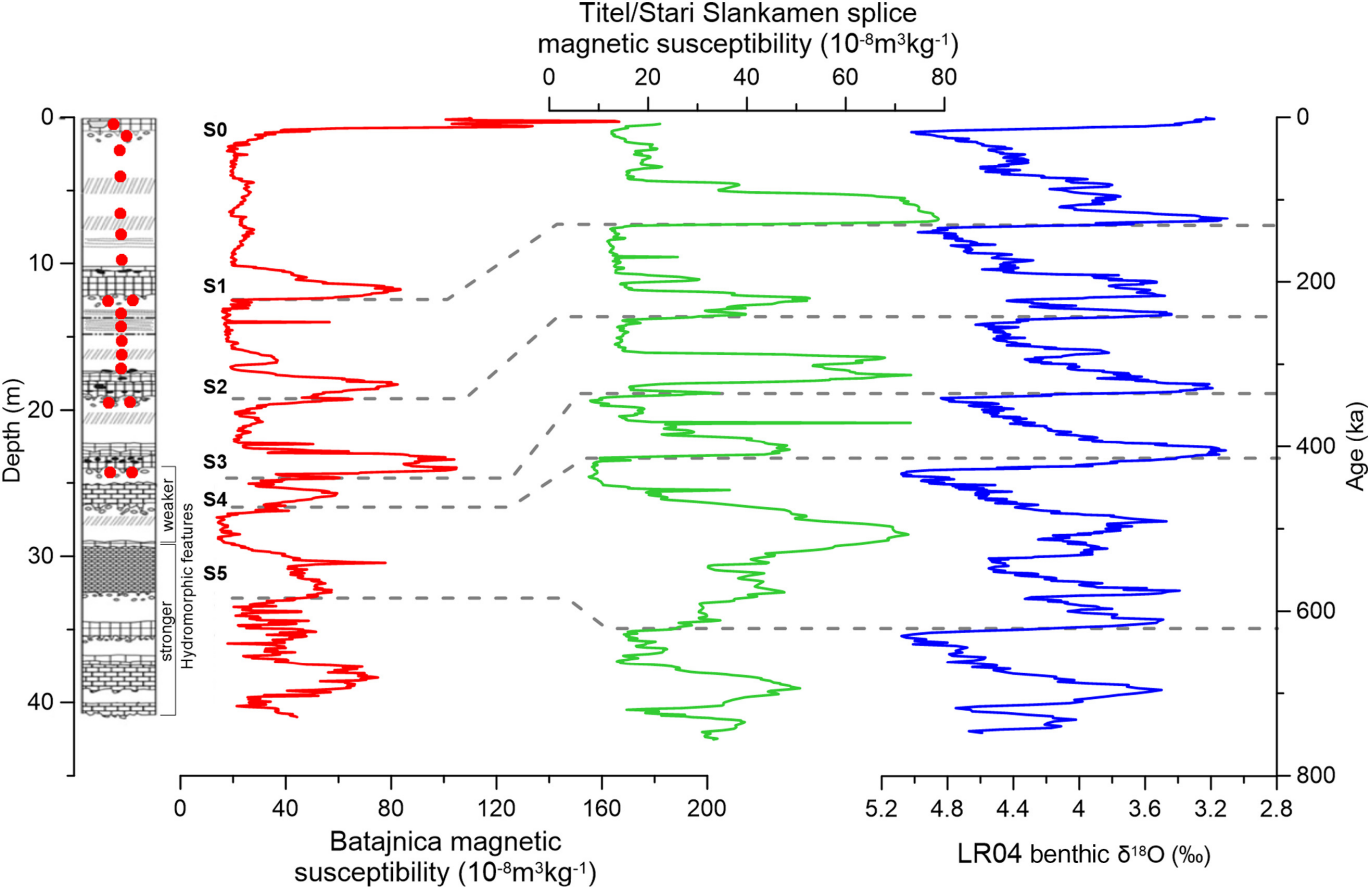
Stratigraphy and magnetic susceptibility of the Batajnica site (Marković *et al*. 2009) and its correlation to the astronomically tuned magnetic susceptibility record from the Titel/Stari Slankamen composite record (Basarin *et al*. 2014) and benthic oxygen isotope stack (Lisiecki & Raymo 2005). Palaeosols are marked from S0 to S5. Dashed lines indicate loess–palaeosol transitions at the Batajnica site and corresponding ages at the Titel/Stari Slankamen composite record and benthic oxygen isotope stack. On the stratigraphical column, the two tephra layers identified in the L2 loess layer are represented with dashed lines.

Batajnica LPS is located along the Danube, near Batajnica settlement, 15 km northwest of Belgrade (latitude 44°55′29″N, longitude 20°19′11″E; Fig. 1). The composite Batajnica LPS previously investigated by Marković *et al*. (2009) reaches a thickness of 40 m. The section comprises at least five loess–palaeosol couplets, extending to or beyond 600 ka (Fig. 2). Two visible dark‐brown (altered) volcanic ash layers have been documented in loess unit L2 (Fig. 2). Previous studies on Batajnica include pedostratigraphical correlations, rubification index and environmental magnetic data analyses (Marković *et al*. 2009). A relative chronology has been obtained by correlating the magnetic susceptibility record with Lingtai (Chinese Loess Plateau) and the SPECMAP oxygen isotope record (Marković *et al*. 2009).

Luminescence investigations were carried out on 18 individual samples collected in stainless steel tubes from the same outcrops studied by Marković *et al*. (2009). Seven samples were taken from the L1 loess unit. Doublet samples were collected from the lower boundaries of the S1, S2 and S3 palaeosols, and one sample from the upper boundary of the S2 palaeosol. Additionally, four samples were taken to assess the chronological range of the L2 tephra layers (Fig. 2).

## Methodology

### Sample preparation and analytical facilities

The luminescence samples were prepared under low‐intensity red light conditions. Gamma spectrometry and water content measurements were carried out using material from the ends of each sample tube, whereas fine quartz (4–11 μm), coarse quartz (63–90 μm) and polymineral fine grains were extracted from the inner part. A treatment with hydrochloric acid (10% concentration) was employed for calcium carbonate removal followed by a treatment with hydrogen peroxide (10% concentration followed by 30%) for organic matter removal. Finer (<63 μm) and coarser (>63 μm) grains were separated through wet sieving. Fine grains (<11 μm) were obtained by settling using Stokes’ law. The quartz fraction was enriched by etching with hydrofluorosilicic acid for 10 days. The 4–11 μm quartz fraction was obtained by centrifugation in distilled water. Polymineral fine grains (4–11 μm) were separated from the finer polymineral fraction obtained after Stokes’ law settling and centrifuging in distilled water (Frechen *et al*. 1996; Lang *et al*. 1996). The coarser (63–90 μm) grain fraction was separated through dry sieving. Since this fraction consists of a polymineral mixture, a density separation using heavy liquid was performed (2.62 and 2.75 g cm^−3^). To isolate the quartz grains from the plagioclase feldspars, a treatment with hydrofluoric acid (40% concentration) was applied for 40 min. A rinse with hydrochloric acid (10%) for 60 min removed the precipitated fluorides. For measurement, fine quartz and polymineral grains were mounted on aluminium discs whereas for coarse grains stainless steel discs were used.

Luminescence measurements were performed using TL/OSL Risø DA‐20 readers, equipped with a classic or automated detection and stimulation head (DASH) (Lapp *et al*. 2015). Luminescence signals were detected by EMI 9235QA and PDM 9107Q‐AP‐TTL‐03 (160–630 nm) photomultipliers. 7.5‐mm‐thick Hoya U‐340 UV filters were used for the detection of the quartz signals while for polymineral fine grain signals a blue filter combination (Schott BG39 + Corning 7‐59, with transmission between 320–460 nm) was used. Laboratory irradiations were carried out using ^90^Sr‐^90^Y radioactive sources that were calibrated with gamma‐irradiated calibration quartz (Hansen *et al*. 2015) for both fine and coarse grain fractions. The dose rate used for measurements was 0.11 Gy s^−1^ for fine grains and 0.13 Gy s^−1^ for coarse grains.

The bleaching experiments on polymineral fine grains were carried out under controlled laboratory conditions using an array of TL29D16/09N lamps, which deliver a power of approximately 1000 W m^−2^ with similar spectral characteristics to natural sunlight (Petrušić *et al*. 2011). We have assumed that 1 day of exposure to this lamp corresponds to 1 day of exposure to natural daylight during summer time.

### Equivalent dose determination

Equivalent doses of fine (4–11 μm) and coarse (63–90 μm) quartz grains were determined using the single‐aliquot regenerative dose (SAR) procedure (Murray & Wintle 2000, 2003). Full details on each protocol are given in Table S1. Optical stimulation was carried out with blue light‐emitting diodes for 40 s at 125 °C. The net CW‐OSL signal used for analysis was integrated over the first 0.308 s of the decay curve and employing an early background subtraction from 1.69–2.30 s interval. Sensitivity changes were corrected by using the OSL response to a test dose of 17 Gy throughout the whole set of measurements. A preheat temperature of 220 °C for 10 s and a cutheat of 180 °C were employed. A high‐temperature bleach of 280 °C for 40 s was performed at the end of each SAR cycle (Murray & Wintle 2003). The robustness of the SAR protocol was checked by the intrinsic performance tests (recycling and recuperation; Murray & Wintle 2003) included in every measurement. OSL IR depletion tests were performed in order to investigate the purity of the quartz luminescence signals on all aliquots measured (Duller 2003). For equivalent dose determination, only the aliquots that yielded recycling and OSL IR depletion ratios within 10% deviation from unity were accepted. Recuperation ratio was considered suitable if it reached <2% of the natural signal.

Two elevated temperature infrared stimulation methods based on the SAR procedure were used for measuring the polymineral fine grains. We first applied a post‐IR IRSL protocol (pIRIR_290_) on all samples from the Batajnica LPS (Table S1b; Buylaert *et al*. 2011b, 2012; Thiel *et al*. 2011a). After a preheat of 320 °C for 60 s, the samples were exposed to IR diodes for 200 s at 50 °C in order to reduce the signal susceptible to fading by allowing recombination of near‐neighbour trap and centre pairs. The signal of interest was recorded during a subsequent IR stimulation at 290 °C for 200 s. A test dose of 17 Gy was used in all measurements, unless otherwise stated. The net signal used for analysis was integrated from the initial ~2.5 s of stimulation minus a background evaluated from the last 50 s. At the end of every cycle, a high‐temperature bleach was performed for 100 s at 325 °C to remove residual charge.

A further post‐IR IRSL protocol (pIRIR_225_) (Table S1c; Roberts 2008; Buylaert *et al*. 2009; Wacha & Frechen 2011; Vasiliniuc *et al*. 2012) was employed to determine equivalent doses on samples previously measured with pIRIR_290_, in order to check the reliability of the pIRIR_290_ ages. After a preheat of 250 °C for 60 s, the aliquots were stimulated with IR diodes, firstly at 50 °C for 200 s and then at 225 °C for 200 s. The signal from the latter stimulation was used in equivalent dose determination. The response to a test dose of 17 Gy was determined in the same way. At the end of each cycle of the protocol, a bleach was performed at 290 °C for 100 s.

### Annual dose determination

High‐resolution gamma spectrometry was applied to determine the radionuclide activity concentrations using a well‐type HPGe detector. In order to achieve ^226^Ra‐^222^Rn equilibrium, the samples were stored for 1 month. The activity of ^238^U was determined indirectly by measuring the ^234^Th emissions of 92.3 and 92.8 keV peaks. The ^226^Ra activity was determined from the emissions at 295 and 351 keV of ^214^Pb and the emission at 609 keV of ^214^Bi. The activity of the ^232^Th was determined indirectly by measuring the following peaks: 338 keV (^228^Ac), 911 keV (^228^Ac), 238 keV (^212^Pb) and 583 keV (^208^TI). For determination of the ^40^K activity, the 1461 keV peak was used. The annual dose rates were determined using the conversion factors tabulated by Guérin *et al*. (2011). The beta attenuation and etching factor for 63–90 μm quartz fraction was assumed to be 0.94±0.05 (Mejdahl 1979). For the 4–11 μm quartz fraction, an alpha efficiency factor of 0.04±0.02 was taken into account whereas for the polymineral fine grains a value of 0.08±0.02 was used (Rees‐Jones 1995). The time averaged water content was assumed to be 15% with a relative error of 25% based on the average humidity of the loess samples in Vojvodina. The external contribution from beta and gamma radiation (additionally alpha radiation for 4–11 μm grains), as well as from the cosmic rays, was included in the total dose rates. For each sample, the dose rate of the cosmic rays was estimated as a function of depth, altitude and geomagnetic latitude (Prescott & Hutton 1994). The internal dose rate contribution for the coarse quartz fraction was assumed to be 0.010±0.002 Gy ka^−1^ (Vandenberghe *et al*. 2008). Given the size of fine grains (4–11 μm), it is assumed that any dose rate derived from internal alpha activity is negligibly small.

Additionally, the total ^210^Pb content was determined for 10 samples by measuring the concentration of ^210^Po (5.304 keV peak, alpha energy) by means of alpha spectrometry. A chemical separation was performed on the loess matrix to isolate the Po isotopes from the other alpha emitters (Data S1). The measurements were carried out using an ORTEC SOLOIST 450 mm^2^ PIPS detector (19 keV resolutions) and an ASPEC‐92 Dual Multichannel analyzer.

### Determination of expected ages for the major palaeosol units based on magnetic susceptibility correlation

Marković *et al*. (2009) described the characteristic stratigraphical pattern in magnetic susceptibility for the uppermost soil and various palaeosol units at Batajnica and how it can be correlated with similar records throughout Eurasia. It is well known that major palaeosols within Eurasian LPSs exhibit a distinctive magnetic enhancement pattern, which is also reflected in grain size and colour proxies, that denotes the degree of pedogenesis, which in turn is hydroclimatically controlled. These patterns and their stratigraphical superposition allow for secure identification of major palaeosols and serve as independent chronostratigraphical markers that constitute the backbone of the loess chronostratigraphy in the wider Danube Basin and beyond (Marković *et al*. 2015; Necula *et al*. 2015). Starting from this well‐established base, Basarin *et al*. (2014) correlated the astronomically tuned magnetic susceptibility record of the Titel/Stari Slankamen composite profile to the benthic oxygen isotope stack (Lisiecki & Raymo 2005) for the last *c. *800 ka.

As such, the major loess–palaeosol transitions identified in Batajnica according to the magnetic susceptibility record were correlated to the corresponding ones in the Titel/Stari Slankamen composite profile (Fig. 2). This approach (see Basarin *et al*. 2014) further enabled a correlation to the benthic oxygen isotope stack (Lisiecki & Raymo 2005). As the luminescence samples were collected from the same outcrop where the magnetic susceptibility samples were taken, previous field marks allowed for secure correlation of our samples and the magnetic susceptibility samples. Expected age estimates have been assigned to samples collected from the Holocene soil and palaeosol boundaries (BAT‐1.0, 1.11, 1.12A, 1.16, 1.17 and 1.19A) based on the data reported by Basarin *et al*. (2014) and their correlation to benthic oxygen ages of Lisiecki & Raymo (2005) (Fig. 2, Table S2).

This method of determining expected ages relies on the assumption that no large erosional gaps occur at the loess–palaeosol transitions for Batajnica and Titel/Stari Slankamen. We are confident that this assumption is valid for the investigated sites as the regional Danube Basin stratigraphy is very well constrained by integrating many profiles; in case a significant erosional gap occurs (e.g. Stari Slankamen L2), it can be clearly identified (Marković *et al*. 2009, 2015). Smaller erosion gaps cannot be excluded, but they are not relevant at the time scale of the uncertainties in the expected depositional ages. Furthermore, in order to account for possible uncertainties, a 10% error has been associated with the boundary ages between loess and soil or palaeosols.

## Results and discussion

### Annual doses and calculation of expected equivalent doses

Tables 1 and S3 contain relevant information on dosimetry and specific radionuclide activities. The annual doses were derived from the activity concentrations of the ^238^U, ^232^Th ^40^K and ^210^Pb measured using gamma spectrometry.

**Table 1:** Summary of the OSL, pIRIR_290_ and pIRIR_225_ ages considering the residual dose estimated based on the modern analogue sample. * indicates the set of polymineral ages considering the residual dose measured after laboratory bleaching. The age uncertainties were determined following Aitken & Alldred (1972). The uncertainties associated with the luminescence and dosimetry data are random; the uncertainties mentioned with the optical ages are the overall uncertainties. The systematic errors taken into account include: 2% beta source calibration, 3% conversion factors, 5% attenuation and etching factors, 3% gamma spectrometer calibration, 15% cosmic radiation, 25% water content. All uncertainties represent 1σ. Specific activities were measured on a well detector by gamma spectrometry and the ages were determined considering 15% water content; beta attenuation and etching factors used for 63–90 μm quartz were 0.94±0.05 (Mejdahl 1979); adopted alpha efficiency factor was 0.04±0.02 for 4–11 μm quartz and 0.08±0.02 for polymineral 4–11 μm fine grains (Rees‐Jones 1995). The total dose rate consists of the contribution from the beta and gamma radiations for coarse grains as well as the contribution from alpha radiation in the case of fine grains. The contribution of cosmic radiation was taken into account and calculated accordingly to Prescott & Hutton (1994). For coarse quartz grains an internal dose rate of 0.01±0.002 Gy ka^−1^ was considered (Vandenberghe *et al*. 2008). In italics are given the equivalent doses calculated by interpolating natural signals onto the saturation region of the dose response curve, and the associated luminescence ages

Sample code	Stratigraphical unit	Depth (cm)	ED (Gy)				Total random error (%)				Total systematic error (%)				Total dose rate (Gy ka^−1^)				Age (ka)				Age (ka)*	
			4–11 μm quartz	63–90 μm quartz	pIRIR_290_ pfg	pIRIR_225_ pfg	4–11 μm quartz	63–90 μm quartz	pIRIR_290_ pfg	pIRIR_225_ pfg	4–11 μm quartz	63–90 μm quartz	pIRIR_290_ pfg	pIRIR_225_ pfg	4–11 μm quartz	63–90 μm quartz	pIRIR_290_ pfg	pIRIR_225_ pfg	4–11 μm quartz	63–90 μm quartz	pIRIR_290_ pfg	pIRIR_225_ pfg	pIRIR_290_ pfg	pIRIR_225_ pfg
BAT 1.0	S0/L1	87	31±1	28±1	36±9	37±6	4.3	4.6	25.2	16.5	8.8	6.7	8.4	8.4	2.9±0.08	2.4±0.07	3.2±0.09	3.2±0.09	11±1	12±1	11±3	12±2	22±2	19±2
BAT 1.1	L1	103	37±1	40±2	43±9	45±6	4.1	5.9	21.2	13.7	8.7	6.8	8.3	8.3	2.9±0.09	2.4±0.07	3.3±0.11	3.3±0.11	13±1	17±2	13±3	14±2	24±2	22±2
BAT 1.7		214	74±1	91±6	89±10	91±7	2.7	7.0	11.5	8.0	9.0	6.8	8.6	8.6	3.1±0.07	2.6±0.06	3.6±0.08	3.6±0.08	24±2	35±3	26±4	26±3	36±4	33±3
BAT 1.8		423	108±1	110±6	121±9	105±11	2.6	6.0	7.8	10.7	9.2	6.9	8.8	8.8	3.5±0.08	2.9±0.07	4.0±0.10	4.0±0.10	31±3	38±3	31±4	27±4	39±4	33±4
BAT 1.9		641	145±3	116±6	185±18	162±10	4.7	6.6	10.7	7.6	8.8	6.9	8.5	8.5	2.9±0.12	2.4±0.10	3.2±0.12	3.2±0.12	51±5	48±5	57±8	50±6	69±8	58±6
BAT 1.10		821	176±2	168±7	217±16	189±10	3.2	5.1	8.0	6.1	8.6	7.0	8.3	8.3	2.8±0.08	2.4±0.07	3.1±0.10	3.1±0.10	62±6	70±6	69±8	60±7	80±8	68±7
BAT 1.11	S1/L1	953	217±4	211±8	328±14	292±23	3.1	4.6	5.0	8.3	8.8	7.0	8.4	8.4	3.4±0.09	2.9±0.07	3.8±0.10	3.8±0.10	64±6	74±6	87±9	77±9	96±9	84±9
BAT 1.12 A	L2/S1	1200	237±3	224±12	490±21	377±19	3.3	6.2	5.3	5.9	9.3	7.0	8.9	8.9	2.6±0.08	2.1±0.06	2.9±0.09	2.9±0.09	93±9	105±10	169±17	130±14	181±18	138±14
BAT 1.12 B		1200	206±4	262±16	408±15	290±13	4.3	7.2	5.3	5.9	9.5	6.9	9.0	9.0	2.7±0.10	2.3±0.08	3.1±0.12	3.1±0.12	76±8	116±12	132±14	94±11	143±15	102±11
BAT 1.13 A	L2	1300	275±8	*307±33*	*743±42*	*572±17*	4.5	11.2	6.6	4.6	8.9	7.0	8.6	8.6	2.5±0.09	2.1±0.07	2.9±0.09	2.9±0.09	108±11	*144*±19	*260*±28	*200*±20	*273*±29	*209*±20
BAT 1.13 B		1300	345±9	*357±46*	*745±41*	*734±33*	4.2	13.3	6.4	5.6	8.7	7.0	8.3	8.3	2.7±0.09	2.3±0.07	3.0±0.10	3.0±0.10	130±13	*159*±24	*252*±27	*249*±25	*264*±27	*257*±26
BAT 1.14 A		1450	334±7	*280±28*	*762±63*	*542±17*	3.6	10.4	8.8	4.3	9.1	7.0	8.7	8.7	2.6±0.08	2.2±0.06	2.9±0.09	2.9±0.09	129±13	*129*±16	*261*±32	*185*±18	*272*±33	*194*±19
BAT 1.14 B		1450	359±11	*291±16*	*843±68*	*661±42*	4.4	6.3	8.7	7.1	9.2	7.0	8.8	8.8	2.7±0.09	2.2±0.07	3.0±0.09	3.0±0.09	134±14	*131*±12	*279*±35	*219*±25	*291*±35	*227*±25
BAT 1.16	S2/L2	1650	348±6	*366±53*	*1137±129*	*690±28*	3.5	14.8	11.7	5.0	9.2	7.0	8.7	8.7	2.9±0.09	2.4±0.07	3.2±0.10	3.2±0.10	122±12	*153*±25	*353*±52	*214*±22	*363*±52	*221*±22
BAT 1.17	L3/S2	1900	383±12	*333±31*	*1119±126*	*629±24*	4.2	9.7	11.6	4.7	8.9	7.1	8.5	8.5	2.7±0.07	2.3±0.06	3.0±0.09	3.0±0.09	143±14	*147*±18	*373*±54	*210*±20	*385*±54	*217*±21
BAT 1.18		1900	386±10	*377±27*	*1012±107*	*532±51*	3.6	7.6	10.9	9.9	8.9	7.1	8.5	8.5	2.7±0.06	2.3±0.05	3.0±0.08	3.0±0.08	145±14	*168*±17	*339*±47	*178*±23	*351*±47	*186*±24
BAT 1.19 A	L4/S3	2400	486±5	*345±23*		*1178±66*	2.7	7.2		6.1	9.3	7.1		8.9	3.2±0.08	2.7±0.07		3.4±0.09	150±15	*128*±13		*322*±35		*328*±35
BAT 1.19 B		2400	475±7	*414±37*		*1244±62*	3.5	9.5		5.9	9.1	7.2		8.7	3.1±0.10	2.6±0.08		3.2±0.11	153±15	*159*±19		*356*±38		*362*±38

We have investigated the secular equilibrium assumption in the ^238^U chain by assessing the ratio between the activity concentration of ^210^Pb measured directly using the 46 keV line and ^226^Ra indirectly measured using the ^214^Pb (352 and 295 keV) and ^214^Bi (609.3 keV) energy peaks. The average ratio calculated for all samples is 0.48±0.03 (Fig. S1). This is caused either by the escape of ^222^Rn during the burial time or by inaccurate gamma spectrometry measurement of ^210^Pb. Thus, the activity concentration of ^210^Pb was determined indirectly using alpha spectrometry by measuring ^210^Po for 10 samples. As seen in Fig. S2, the ratios between the alpha and gamma spectrometry results are consistent with unity, showing that the activity concentrations of ^210^Pb obtained by gamma spectrometry are accurately determined. This indicates that ~50% radon loss occurs in the investigated loess samples. Thus, the annual doses that consider the measured ^210^Pb were used in to determine the luminescence ages.

By multiplying the expected age estimates (Table S2) by the corresponding environmental dose rate, expected equivalent doses and their associated uncertainties were determined (Table S2).

### Luminescence properties – quartz

For both quartz fractions the net signals displayed a rapid decay during optical stimulation as documented for sample BAT‐1.10 in Fig. S3. The patterns of the natural and regenerative decay curves were found to be similar to that shown by the luminescence signal of the calibration quartz, which is recognized as being dominated by the fast component (Hansen *et al*. 2015). Representative laboratory growth curves are shown in Fig. S3, displaying a good behaviour in the SAR protocol. The dose response curves were well fitted using the sum of two saturating exponential functions. The recycling and IR depletion ratios were within 10% from unity, demonstrating that the sensitivity changes during repeated SAR cycles are accurately corrected for, and the quartz signals are pure (Table S4).

#### Preheat plateau

The dependency of the equivalent doses on the preheat temperature was investigated for sample BAT‐1.13B (fine quartz). Different preheat temperatures ranging from 180 to 280 °C were applied on sets of five aliquots for each sample, in combination with a test dose cutheat of 180 °C. Figure S4 shows that over the investigated interval of preheat temperatures, equivalent doses do not display systematic variation. The recycling ratio and recuperation were satisfactory for all the aliquots measured.

#### Dose recovery

**Figure 3:**
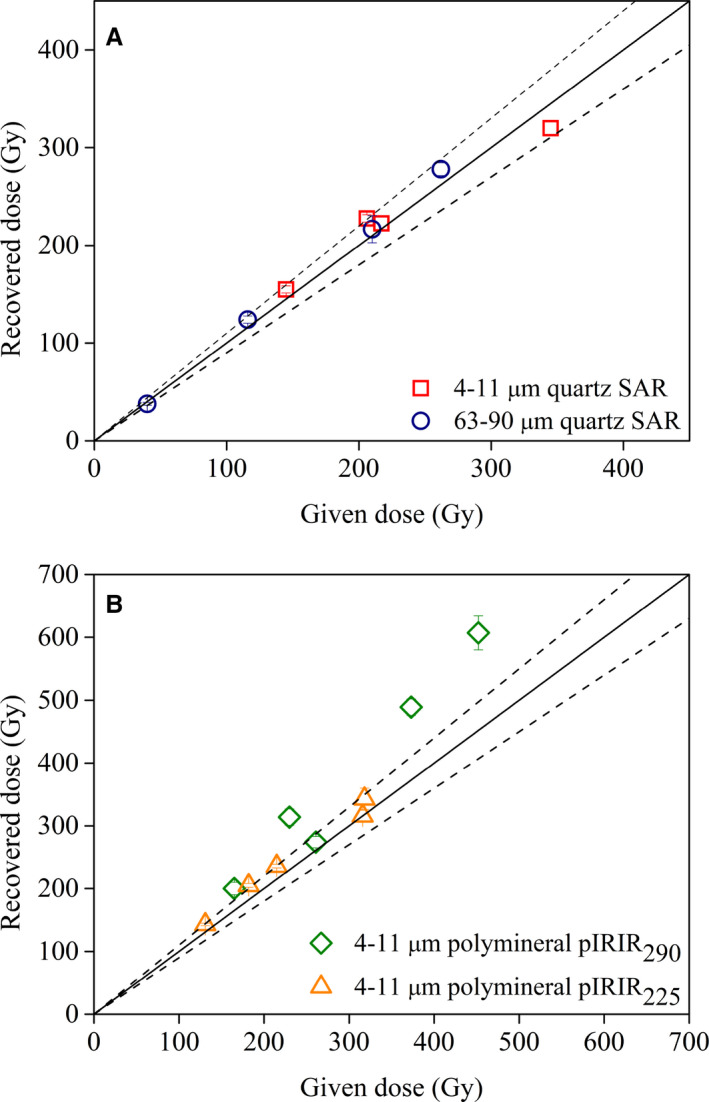
Dose recovery test results for (A) fine (open squares) and coarse (open circles) quartz, (B) polymineral fine grains on pIRIR_290_ (open diamonds) and pIRIR_225_ (open triangles). The given irradiation doses were chosen to match the equivalent dose of each sample. The solid line indicates the ideal 1:1 dose recovery ratio while the dashed lines bracket a 10% variation from unity.

Further investigation was focused on analysing whether the SAR protocol can successfully measure a known irradiation dose prior to any thermal treatment by applying the dose recovery test (Murray & Wintle 2003). Sets of five aliquots were prepared from 4 to 11 μm quartz samples (BAT‐1.9, 1.11, 1.12B, 1.13B) and 63–90 μm quartz samples (BAT‐1.1, 1.9, 1.11, 1.12B). The aliquots were irradiated with a beta dose, chosen to approximate the equivalent doses, after their natural signals were bleached by a repeated exposure to the blue light‐emitting diodes for 100 s at room temperature with a pause of 10 ks. These doses were then determined using the SAR protocol. As seen in Fig. 3A, for the quartz samples, the SAR protocol can successfully recover laboratory doses up to 320 Gy on fine quartz and 260 Gy on coarse quartz.

#### Quartz equivalent doses

Equivalent doses were measured on at least 10 aliquots of each grain size. Tables 1 and S4 summarize the equivalent dose results, as well as the results from the SAR‐intrinsic tests (Recycling, IR depletion and Recuperation).

Equivalent doses measured on fine quartz aliquots range from 31±1 Gy for sample BAT‐1.0 collected from the Holocene soil to 486±5 Gy for sample BAT‐1.19A from below the S3 palaeosol. The natural signal emitted by this sample interpolates well below the laboratory saturation threshold. Yet, the measured fine quartz equivalent dose reaches only 50% of the expected burial dose (Table S2). Coarse quartz equivalent doses range from 28±1 Gy for the sample from modern soil to 262±16 Gy for a sample collected below the S1 palaeosol. For older samples, the natural signal was in field saturation and close to laboratory saturation (see below).

### Luminescence properties – polymineral fine grains

Figure S3C, D shows representative growth curves for sample BAT‐1.10 constructed using pIRIR_290_ and pIRIR_225_ protocols. The dose response curves were well fitted using the sum of two saturating exponential functions. The growth curves pass very close to the origin demonstrating that the recuperation is insignificant. As can be seen in insets of Fig. S3C, D the decays with stimulation time of the natural and regenerative signals have similar shapes.

#### Effect of test dose size on equivalent dose

**Figure 4:**
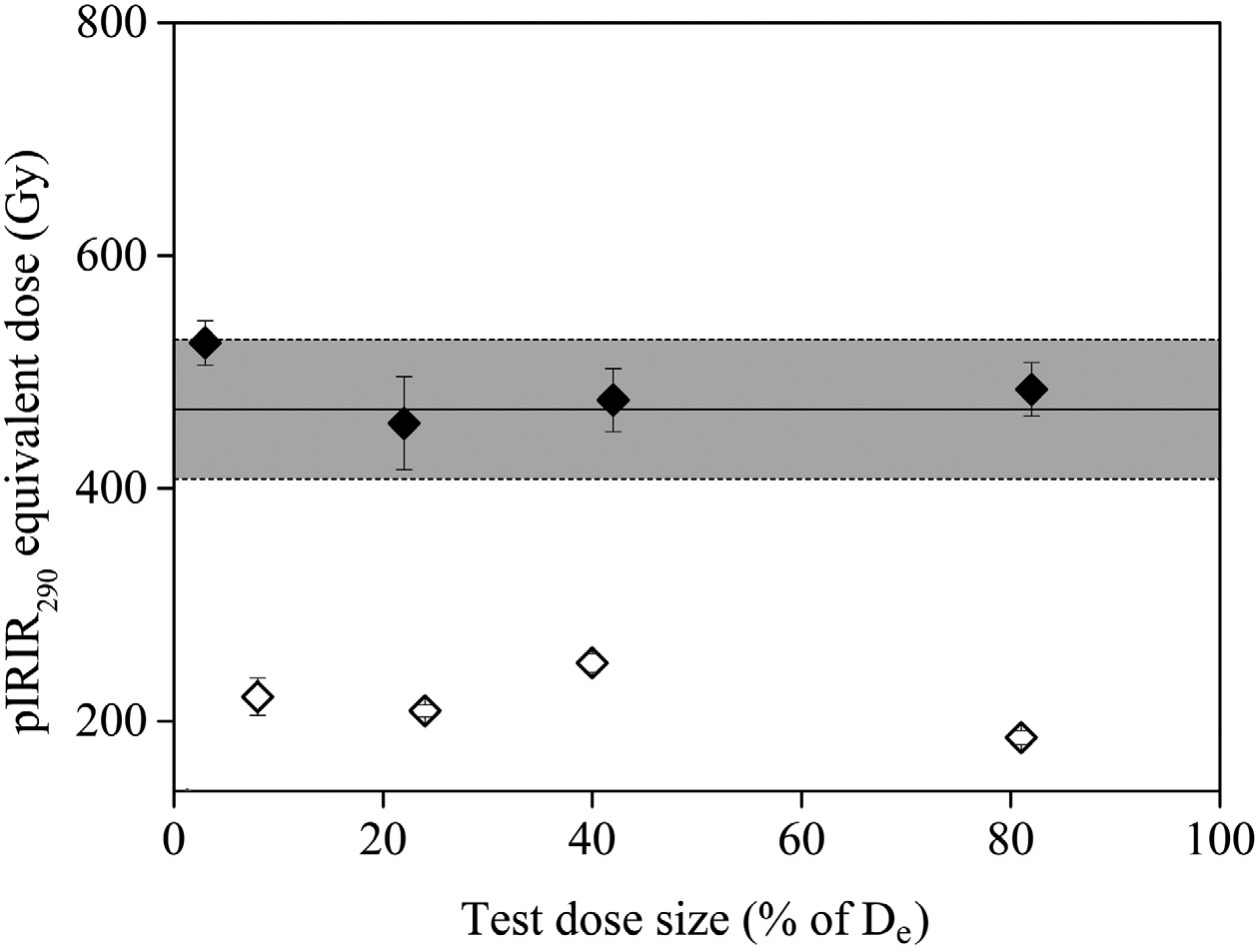
Dependence of measured pIRIR_290_ equivalent dose on the size of test dose for samples BAT‐1.9 (open symbols) and BAT‐1.12A (filled symbols). The expected equivalent doses for sample BAT‐1.12A, taken from Table S2, are highlighted with grey. Solid lines are used to indicate the expected values while error ranges are given by dashed lines.

The effect of test dose size on the measured equivalent dose using the pIRIR_290_ protocol was investigated on samples BAT‐1.9, 1.12A and 1.16 (Fig. 4). Sets of three to five aliquots were used for each test dose (except for the 17 Gy test dose). As shown in Fig. 4 for samples BAT‐1.9 and BAT‐1.12A no systematic trend in equivalent dose values with test dose magnitude can be seen. Although not so clear, this is also valid for sample BAT‐1.16, which yielded pIRIR_290_ equivalent doses of 1172±129 and 996±64 Gy by employing test dose sizes of 2 and 50% of the D_e_, respectively. The expected equivalent dose for sample BAT‐1.16 is 617±84 Gy (Table S2). It is important to note that the natural signal emitted by sample BAT‐1.16 normalized to a test dose of 17 Gy was interpolated in the saturation region of the dose response curve. The results from the Batajnica loess samples indicate that the dependence of the equivalent dose on the size of test dose, supported by investigations in Chinese loess (Yi *et al*. 2016), might not be a general phenomenon.

#### Determination of residual doses

One of the most important assumptions of the luminescence dating method is that the luminescence signal of the mineral has been fully reset in nature by light exposure prior to deposition. The aeolian nature of loess deposits suggests us that the mineral constituents have experienced considerable daylight exposure. Buylaert *et al*. (2012) showed that PIRIR signals bleach at a much slower rate than the quartz OSL signal. For pIRIR_225_ signals emitted by K‐feldspar residual doses not exceeding 2 Gy were reported (e.g. Buylaert *et al*. 2009). But later on, hard‐to‐bleach (or un‐bleachable) pIRIR signals amounting to ~6 Gy have been reported for K‐feldspar extracts from Chinese loess after extensive exposure in a solar simulator (Yi *et al*. 2016). Bleaching experiments conducted by Stevens *et al*. (2011) on polymineral fine grains from loess collected from the Carpathian Basin suggest that the value of the residual is influenced by the exposure time and conditions as well as the equivalent dose of the sample with residual values for pIRIR_290_ signals ranging from 5.5 Gy (for 28 days exposure outside of a sample taken from the S0/L1 boundary) to 33 Gy (in the case of a sample taken from L2 that was exposed to window light for 8 days).

Besides conducting laboratory bleaching experiments another way to derive the residual doses is by measuring a modern analogue, which is a modern sample. Buylaert *et al*. (2011a) carried out such a study on three samples of modern dust and three samples with equivalent doses of <1 Gy collected from the Chinese Loess Plateau. A residual average dose of 4 Gy was obtained for pIRIR_225_ signals while a higher average value of 11 Gy was reported in the case of pIRIR_290_ signals.

Due to the complexity of bleaching behaviour of feldspar signals, as explained above, in this study we conducted residual doses corrections both by laboratory measurements of the residuals and by using the modern analogue approach.

Three to five aliquots were used for residual dose measurement. Fresh discs were exposed to the UV lamp for 1 month before the dose measurements. The measured residual values amount to a maximum of 0.5 and 4% of the measured pIRIR_225_ and pIRIR_290_ equivalent doses, respectively (Tables S5, S6). Similar pIRIR_225_ residual doses (after laboratory bleaching) have been reported by Wacha & Frechen (2011) for loess samples collected from the Gorjanović site near Vukovar, Croatia. Our pIRIR_290_ residual results are consistent with those obtained by Constantin *et al*. (2019) for loess samples from Mošorin (Vojvodina, Serbia) and Veres *et al*. (2018) for Stayky LPS, Ukraine. It is important to note that in this study the size of the residual doses does not vary with age.

As there was no true modern analogue at our disposal, we estimated the values for the residuals by using the young samples where age control is available as follows. We estimated the residual doses by comparing the polymineral ages with the average fine and coarse quartz age of the uppermost sample BAT‐1.0. Quartz ages obtained for this sample are in excellent agreement with independent age control (11.5±1 ka, Table S2) based on magnetic susceptibility correlation. The age offset was converted into residual dose using the annual dose rate determined for each sample. These residual doses were subtracted from all the pIRIR D_e_ values prior to calculation of the ages (Table 1).

#### Dose recovery

Dose recovery tests (Murray 1996; Wallinga *et al*. 2000) were performed on five aliquots from samples BAT‐1.8 to 1.11 and BAT‐1.12B. The natural signal was bleached by exposing fresh aliquots to a lamp for 2 weeks. Laboratory known doses were chosen to match the measured equivalent dose. The residual doses were subtracted from the recovered doses before calculating the dose recovery ratios. Unless otherwise stated, a test dose of 17 Gy was used throughout the measurements. As shown in Fig. 3B the pIRIR_225_ protocol can successfully recover laboratory doses up to 316 Gy, while the pIRIR_290_ protocol systematically overestimates the given laboratory doses. Following the suggestions of Yi *et al*. (2016), we carried out pIRIR_290_ dose recovery tests using higher test doses. For sample BAT‐1.9, the dose recovery ratio improved from 1.36±0.06 to 1.07±0.03 using a 17 Gy test dose (7% of D_e_) and a 50 Gy test dose (23% of D_e_), respectively. However, for sample BAT‐1.11 poor dose recovery ratios of 1.31±0.04 and 1.41±0.10 were obtained by using test doses of 17 Gy (5% of D_e_) and 150 Gy (41% of D_e_), respectively. As shown above, the size of the test dose does not influence the measured equivalent dose (Fig. 4) or the saturation characteristics of the pIRIR_290_ dose response curves (see below). Thus, in this paper we will discuss the equivalent doses measured with a test dose of 17 Gy.

Unsatisfactory dose recovery tests for pIRIR_290_ have also been reported by Murray *et al*. (2014) for Serbian loess, by Veres *et al*. (2018) for Ukrainian loess and by Thiel *et al*. (2011b) for Japanese loess. On fluvial and lacustrine sediments from Heidelberg Basin, Germany, Li *et al*. (2018) reported poor pIRIR_290_ dose recovery and good pIRIR_225_ dose recovery. However, such behaviour is not general since Thiel *et al*. (2011a), Yi *et al*. (2016), Bösken *et al*. (2017), Stevens *et al*. (2018) and Zhang *et al*. (2018) reported good pIRIR_290_ dose recovery ratios for the loess in the Middle Danube basin and the Chinese Loess Plateau.

#### Polymineral fine grains equivalent doses

The pIRIR_290_ and pIRIR_225_ equivalent doses after the subtraction of residual doses are displayed in Table 1 while the measured equivalent doses are presented in Table S4. The pIRIR_290_ equivalent doses vary from 36±9 Gy (BAT‐1.0) to 490±21 Gy (BAT‐1.12A). The smallest pIRIR_225_ equivalent dose is 37±6 Gy (BAT‐1.0), whereas the maximum is 377±19 Gy (BAT‐1.12A).

The natural pIRIR_225_ and pIRIR_290_ signals yielded by older samples scatter around or above the saturation threshold of the dose response curve (see below).

#### Fading rate measurements for high doses

While pIRIR_225_ fading rates >1%/decade are reported in the literature (Thomsen *et al*. 2008; Buylaert *et al*. 2009; Zhang *et al*. 2018), Vasiliniuc *et al*. (2012) calculated fading rates ranging from 0.6 to 1.3%/decade on samples collected from the Last Glacial loess in Mircea‐Voda, Romania, and concluded they are laboratory artefacts. Stevens *et al*. (2011), Thiel *et al*. (2011a) and Murray *et al*. (2014) reported fading rates considered to be laboratory artefacts for polymineral pIRIR_290_ signals on Serbian and Austrian loess sites along the Danube valley. Balescu *et al*. (in press) determined extremely low fading rates (<0.18%/decade) for pIRIR_290_ signals emitted by K‐feldspars extracted from Bulgarian loess.

Fading rates (percentage of the signal lost per decade of time; Aitken 1985) were measured on fresh 4–11 μm polymineral aliquots from samples BAT‐1.11 and BAT‐1.19A (Fig. S5, Table S7). The aliquots were bleached by exposure to a lamp for 2 weeks and irradiated with beta doses approximating the equivalent doses for BAT‐1.11 (317 and 364 Gy) and BAT‐1.19A (400 Gy). A test dose of a magnitude of 17 Gy was used throughout all fading measurements. Four consecutive prompt read‐outs were carried out before fading measurement. The aliquots were preheated before storage. Following the read‐out after different storage times, two consecutive prompt read‐outs were carried out.

Figure S5A–L shows a significant scatter in the pIRIR sensitivity‐corrected luminescence signals registered during prompt read‐outs. It is interesting to note that the signal intensity recorded after the first instantaneous read‐out always lies above the values recorded during the subsequent prompt read‐outs. We consider that no systematic variation with delay time is identified for the pIRIR sensitivity‐corrected luminescence signals.

The luminescence stability of quartz is well known, and previous studies reported that the quartz signal is not thought to suffer from anomalous fading (Aitken 1998). However, Thiel *et al*. (2011a) reported fading rates of 1.3±0.3%/decade for fine quartz extracted from Chinese loess. Fading tests were also performed by Lowick & Valla (2018) on fine and coarse quartz. They determined quartz *g*‐values ranging from 0 to 6%/decade while for calibration quartz *g*‐values of 1–2%/decade were measured.

We performed fading measurements on 4–11 μm quartz and 63–90 μm quartz extracted from sample BAT‐1.11 in order to test whether the calculated *g*‐values on polymineral material are fitting artefacts caused by large scatter in the data set. The aliquots were bleached twice with blue LEDs for 100 s at room temperature. Between the two stimulations, a pause of 10 ks was used. After bleaching, the *g*‐values were measured using the SAR‐OSL protocol in the same manner as for the polymineral fine grains. Figure S5M–S shows that the scatter in the quartz data is comparable with the polymineral data set. We report average *g*‐values of 2.49±0.44 and 2.92±0.61%/decade for 4–11 and 63–90 μm quartz, respectively (Table S7). Such *g*‐values cannot reflect real fading as this would imply a significant signal loss during burial. This is in contradiction with our observation that the natural signals emitted by 63–90 μm quartz are close to full saturation, as discussed below.

We interpret the presented data set as follows. There is no solid evidence that quartz and pIRIR_225_ and pIRIR_290_ polymineral signals fade; rather it appears that by using this procedure on the investigated samples, one cannot measure reliable g‐values. Since we cannot confidently measure *g*‐values using the short‐term standard fading measurement procedures in the following we discuss the uncorrected pIRIR_225_ and pIRIR_290_ ages for samples BAT‐1.0–BAT 1.12B. One should also note that fading corrections should not be attempted if the natural signal interpolates beyond the linear range of the dose response curve, as is the case with most of the natural signals emitted from the Batajnica samples (Huntley & Lamothe 2001).

#### High laboratory doses given on top of naturally accrued doses

Previous studies reported a mismatch between SAR dose response curves and the dose response curve constructed when large doses were added on top of natural ones for fine and coarse quartz, indicating that the SAR‐OSL protocol is problematic in the high dose range. Anechitei‐Deacu *et al*. (2018) showed that in the case of fine quartz extracted from aeolianites the sensitivity‐corrected luminescence signal after a large dose was added on top of the natural one was not found to be at the same level to the laboratory saturation region, reaching only 86% of SAR laboratory full saturation intensity, while in the case of coarse quartz 96% of full saturation was attained. For quartz extracted from Ukrainian loess, Veres *et al*. (2018) showed that after adding 3500 Gy on top of the natural signal of 4–11 μm quartz (De ~550 Gy), the sensitivity‐corrected signal reached 96% of the luminescence level induced by a 4000 Gy beta dose. In the case of the 63–90 μm fraction identical luminescence levels were measured after adding a 500 Gy beta dose on top of the natural signal (D_e_ ~500 Gy) and after a 1000 Gy beta dose.

If large laboratory doses are added on top of natural signals found in field saturation, an increase of the signals may suggest a degree of fading that may have been undetected during standard fading rates measurements. On the other hand, if a large dose is given on top of the naturally accrued doses, one normally expects the magnitude of the measured signal to be the same as the saturation value measured in the SAR protocol.

We chose to perform such an experiment on sample BAT‐1.19A, collected below the S3 palaeosol. As can be seen in Fig. 7A,, for a sample with an expected age of more than 300 ka, the pIRIR_225_ natural signal reaches 84±1 % of saturation level, while the pIRIR_290_ natural signal lies above the laboratory dose response curve (105±3%). For quartz, natural sensitivity‐corrected signals are at 95±6% of laboratory saturation levels in the case of 63–90 μm grains, while in the case of fine grains only 56±1% of saturation level is reached.

Ten aliquots of these samples were prepared for each measurement protocol. The first five aliquots were used as control aliquots for recording the natural signals (L_n_/T_n_), while the rest of the aliquots were irradiated to a beta dose added on top of the naturally accrued dose. The magnitude of this dose was chosen to be large enough for the signal to reach full saturation (Fig. 7A). The sensitivity‐corrected luminescence signal (denoted as (L_n_/T_n_)*) was recorded after the large dose was added. The same aliquots were subsequently measured in a standard SAR protocol after an irradiation to a dose of 5000 Gy, chosen to equal the equivalent dose plus the given dose. The value of this normalized signal is hereafter denoted as (L_x_/T_x_).

If (L_n_/T_n_)* is greater than (L_n_/T_n_) there is the possibility that during burial time some loss of the signal may have occurred. In addition, if (L_n_/T_n_)* is greater or smaller than (L_x_/T_x_), issues regarding unknown measurement problems during the first cycle of the measurement or other as yet unknown causes can also be suspected.

From Table S8 it can be seen that for quartz the ratio between (L_n_/T_n_)* and (L_n_/T_n_) is 1.90±0.02 in the case of fine grains and 0.96±0.08 in the case of coarse grains. For polymineral fine grains the ratio of (L_n_/T_n_)* to (L_n_/T_n_) is 1.35±0.03 in the case of pIRIR_225_ and 1.20±0.04 in the case of pIRIR_290_. The ratio between (L_n_/T_n_)* and (L_x_/T_x_) in the case of pIRIR_225_ is 1.10±0.03 while for pIRIR_290_ is 1.15±0.05. In the case of fine and coarse quartz corresponding ratios of 0.95±0.02 and 1.03±0.11, respectively, were obtained (Table S8).

This indicates that in the case of all signals excepting coarse quartz OSL the maximum light levels attained during prolonged natural irradiation can be increased by additional laboratory irradiation. We choose to restrain ourselves from interpreting this observation as an indicator of signal fading during burial because our results here as well as those obtained in fading tests indicate that it is likely that the signals measured during the first measurement cycle in pIRIR protocols slightly overestimate the response obtained for the same dose later given in the SAR protocol. The cause of this behaviour needs further investigation.

### Luminescence ages

The ages calculated using the measured activity concentrations of ^210^Pb are generally 10 to 20% older than those calculated assuming secular equilibrium in the ^238^U chain, but the two sets of ages are consistent within error limits (Fig. S6). Assuming a constant radon loss over the whole sample burial time, the set of ages calculated using the measured activity concentrations of ^210^Pb is discussed in the following section.

Table 1 displays the 4–11 μm quartz, 63–90 μm quartz, pIRIR_290_ and pIRIR_225_ polymineral ages assumed to be unaffected by fading and by considering two possibilities for residual dose correction, namely by using the values obtained by laboratory bleaching experiments and by considering residuals derived through the use of a modern analogue. Only the ages obtained by using the latter correction procedure are discussed further on.

For Batajnica, the luminescence ages are in agreement with the previous chronological framework based on magnetic susceptibility correlation to other records (Buggle *et al*. 2009; Marković *et al*. 2009), placing the L1 loess unit into the Last Glacial.

**Figure 5:**
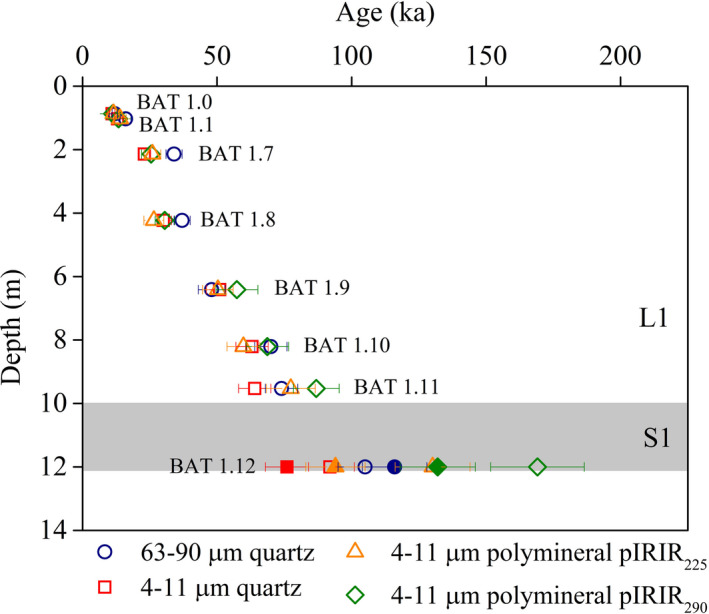
Quartz OSL and polymineral pIRIR_225_ and pIRIR_290_ ages for the uppermost loess–palaeosol alternation at the Batajnica site. Note the doublet samples collected at 12 m depth: open symbols indicate sample BAT‐1.12A while filled symbols represent sample BAT‐1.12B.

**Figure 6:**
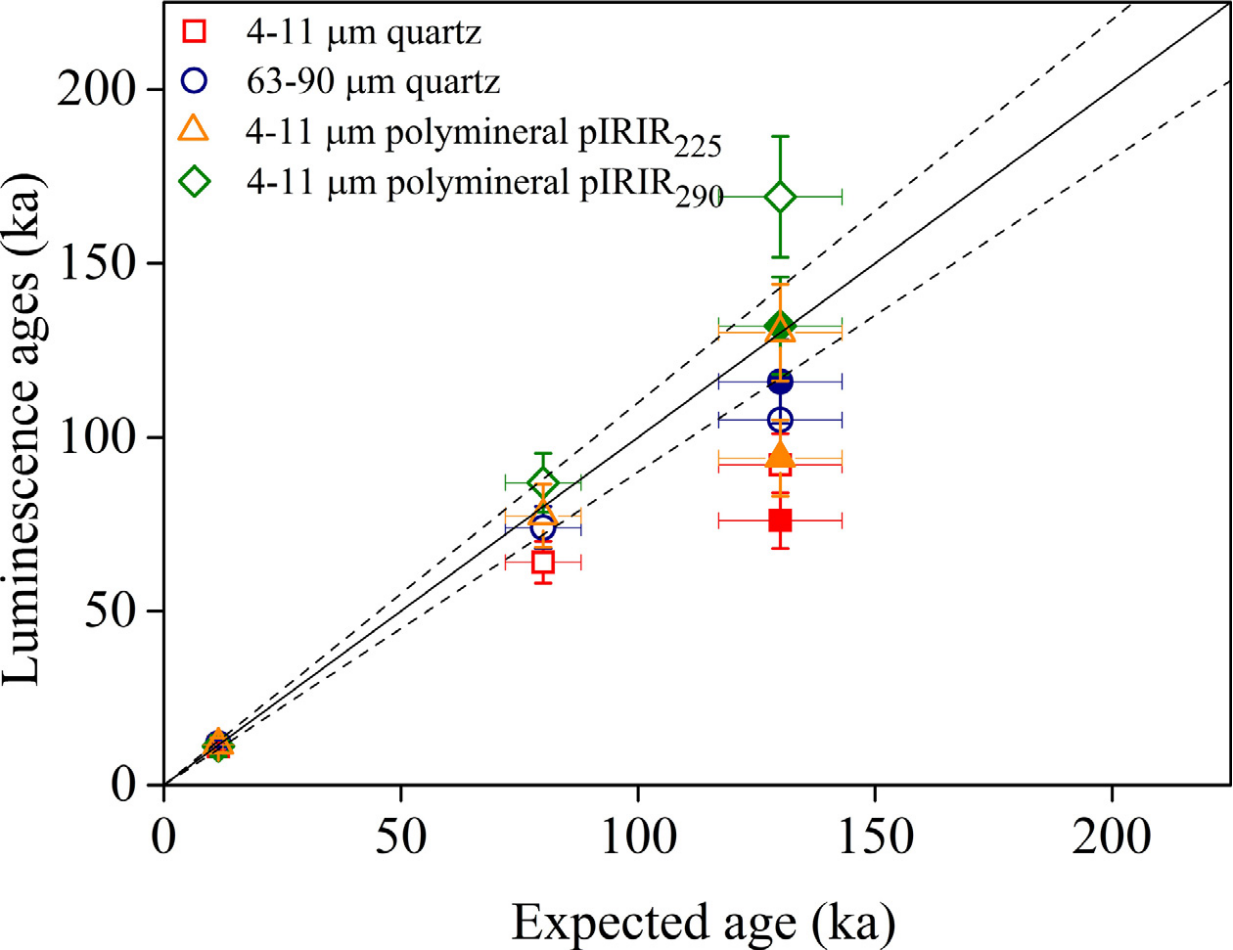
Luminescence ages plotted against expected ages for samples BAT‐1.0, BAT‐1.11, BAT‐1.12A and BAT‐1.12B (filled symbols). The expected ages are derived from the correlation between loess–palaeosol boundaries and benthic isotope stack (Fig. 2, Table S2).

Except for sample BAT‐1.9, all fine quartz ages are systematically lower than coarse quartz ages and the difference between them increases with depth (Fig. 5), with the fine quartz underestimating the expected depositional ages based on magnetic stratigraphy (Fig. 6). At the neighbouring Titel loess section, Perić *et al*. (2019) reported unreliable fine quartz ages beyond 30–35 ka (~100 Gy).

The coarse quartz, polymineral pIRIR_225_ and pIRIR_290_ ages largely agree (Fig. 5) and within errors are consistent with the expected ages (Fig. 6).

Immediately below the S1 palaeosol, the coarse quartz, pIRIR_225_ and pIRIR_290_ yielded ages up to 116±12 ka (262±16 Gy), 130±14 ka (377±19 Gy) and 169±17 ka (490±21 Gy), respectively. However, it is difficult to interpret these results obtained immediately below the S1 palaeosol since the doublet samples BAT‐1.12A, B, differ by 23% on fine quartz, 10% on coarse quartz, 38% on pIRIR_225_ and 28% on pIRIR_290_. This causes a difference of 17 and 11 ka on fine and coarse quartz, respectively, and of 36 ka on polymineral fine grains. As the dosimetry data for the doublet samples are consistent, with the exception of ^210^Pb, the age discrepancies seem to originate from the difference between the equivalent doses. More investigations are required to explain the different equivalent dose results yielded by doublet samples.

For samples taken from deeper units (BAT‐1.13A to 1.19A, B), except for fine quartz, the natural signal is in the region of laboratory saturation. Interpolation of the natural signal onto the high curvature region of the dose response curve results in large and asymmetric uncertainty in the equivalent dose. Consequently, the obtained luminescence ages for samples BAT‐1.13A to 1.19A, B have low precision and their reliability is questionable in terms of accuracy, as is shown below.

### Accuracy of the reported luminescence ages

In order to identify the factors that control the upper limit of luminescence dating we investigate the saturation characteristics of the natural and laboratory signals emitted by both quartz and polymineral grains.

#### Laboratory dose response curves

**Figure 7:**
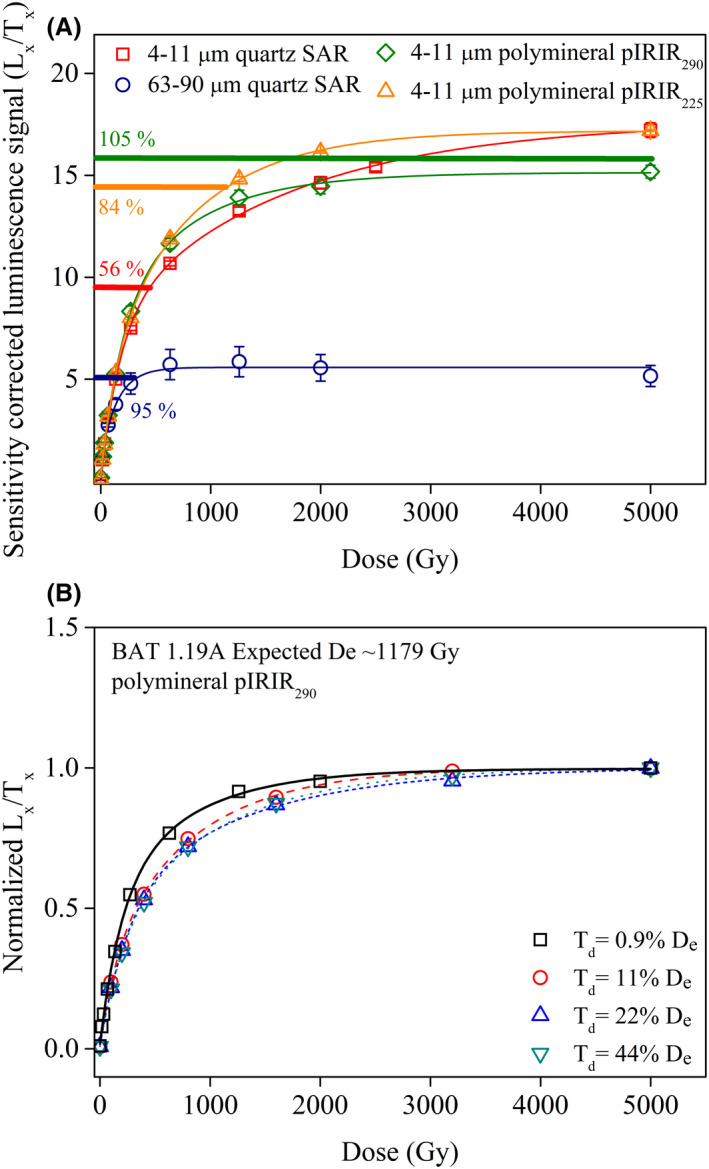
A. Laboratory dose response curves constructed for 4–11 μm and 63–90 μm quartz, 4–11 μm polymineral grains measured with pIRIR_225_ and pIRIR_290_ protocols on sample BAT‐1.19A (>300 ka). All growth curves were fitted with the sum of two saturating exponential functions. Each datapoint represents an average of three measurements. Horizontal lines indicate the average natural sensitivity‐corrected luminescence signals (L_nat_/T_nat_). The average L_nat_/T_nat_ obtained on all aliquots measured, including D_e_ measurements, is interpolated on the growth curve. The percentage indicated for each interpolation represents the ratio between the L_nat_/T_nat_ and the L_x_/T_x_ measured for the 5000 Gy regenerative dose. B. pIRIR_290_ laboratory dose response curves constructed on sample BAT‐1.19A with different test doses: 17 Gy (0.9% D_e_), 200 Gy (11% D_e_), 400 Gy (22% D_e_) and 800 Gy (44% D_e_). Three aliquots were measured for each test dose. All growth curves were fitted with the sum of two saturating exponential functions (*I* = *I*_0_ + *a*·(1 − exp(−*D*/*D*_01_)) + *c*·(1 − exp(−*D*/*D*_02_)). The corrected luminescence signals have been normalized to the sum of the amplitude of the exponential functions obtained by fitting (*a *+ *c*).

Laboratory dose response curves up to 5000 Gy were constructed on sample BAT‐1.19A collected from below the S3 palaeosol (>300 ka according to Marković *et al*. 2009) using 4–11 and 63–90 μm quartz as well as polymineral fine grains employing the pIRIR_225_ and pIRIR_290_ protocols (Fig. 7A). All dose response curves were best fitted with the sum of two saturating exponential functions:

(1) $$I=I_{0}+a\left(1-e^{\frac{-D}{D_{01}}}\right)+c\left(1-e^{\frac{-D}{D_{02}}}\right)$$

where *I* is the intensity of the signal for a given dose *D*,* I*_0_ is the intercept, *a* and *b* are the amplitudes of the exponential functions while *D*_01_ and *D*_02_ are the doses that characterize the onset of saturation of each exponential function, also named characteristic doses in the literature. Following the suggestion of Wintle & Murray (2006) we consider the saturation threshold at 85% of the laboratory dose response curve. We assess the closeness to saturation of natural signals by calculating the ratios between the average sensitivity‐corrected natural signals (L_nat_/T_nat_) and the corrected luminescence signals measured for the 5000 Gy regenerative dose (L_x_/T_x 5000 Gy_).

The laboratory dose response curve on 63–90 μm quartz saturates much earlier than fine quartz and polymineral samples and has characteristic saturation doses of *D*_01_ = 21±28 Gy and *D*_02_ = 153±46 Gy. The coarse quartz natural signal reaches laboratory saturation. Fine quartz on the other hand, yields a laboratory luminescence signal that continues to grow to doses beyond 5000 Gy and the natural signal is interpolated well below the saturation region of the dose response curve. However, the equivalent dose measured for this sample underestimates severely the expected equivalent dose (Table S2). The characteristic doses for fine quartz dose response curves are *D*_01_ = 178±17 Gy and *D*_02_ = 1635±164 Gy. Timar‐Gabor *et al*. (2012) also found the natural fine quartz signal of an infinitely old sample from Costinesti LPS (Romania) not to be in saturation. Timar‐Gabor *et al*. (2017) determined similar saturation characteristic doses for worldwide loess and samples of various sedimentary origins.

It is important to note that pIRIR_225_ and pIRIR_290_ laboratory dose response curves have lower characteristic doses than the fine quartz. We report *D*_01_ = 142±13 Gy and *D*_02_ = 795±36 Gy for pIRIR_225_ while for pIRIR_290_ *D*_01_ = 193±32 Gy and *D*_02_ = 764±145 Gy. The natural pIRIR_225_ signal of sample BAT‐1.19A is 84±0.7% of laboratory saturation while the natural pIRIR_290_ signal is 105±3% and lies slightly above the light level corresponding to the 5000 Gy dose (Fig. 7A, Table S9).

Previous studies document the influence of the magnitude of the test dose on the pIRIR_290_ laboratory growth curves up to high doses (e.g. Murray *et al*. 2014; Yi *et al*. 2016; Colarossi *et al*. 2018). Figure 7B shows pIRIR_290_ laboratory dose response curves up to 5000 Gy on sample BAT‐1.19A using different test doses: 200 Gy (11% of De), 400 Gy (22% of De) and 800 Gy (44% of De). The test doses can be expressed as a fraction of light saturation derived from the growth curve, and ratios of 0.37±0.01, 0.53±0.01 and 0.72±0.01 are determined for 200, 400 and 800 Gy, respectively. The growth curves were fitted with the sum of two saturating exponential functions using Equation (1). The corrected luminescence signals were normalized to the sum of the values of the amplitudes of the exponential functions obtained by fitting (a+c). The saturation parameters (D_01_; D_02_) do not display a significant increase with increased magnitude of test dose (Table S10). Based on our finds for Batajnica, we conclude that the dependence of the saturation parameters on the size of the test dose reported for Chinese loess (Yi *et al*. 2016) or fluvial sediments from South Africa (Colarossi *et al*. 2018), might not represent a general feature of pIRIR_290_ laboratory signals.

In order to quantify the closeness of the natural sensitivity‐corrected luminescence signals to laboratory saturation for all samples collected from the deeper units (BAT‐1.12A‐BAT‐1.19B), we calculated the ratio between the natural sensitivity‐corrected luminescence signal and the average maximum corrected luminescence signals induced by a dose of 5000 Gy. The latter was measured after constructing laboratory dose response curves up to 5000 Gy on samples BAT‐1.17 and BAT‐1.19A. The ratios are given in Table S11.

#### Natural dose response curves

**Figure 8:**
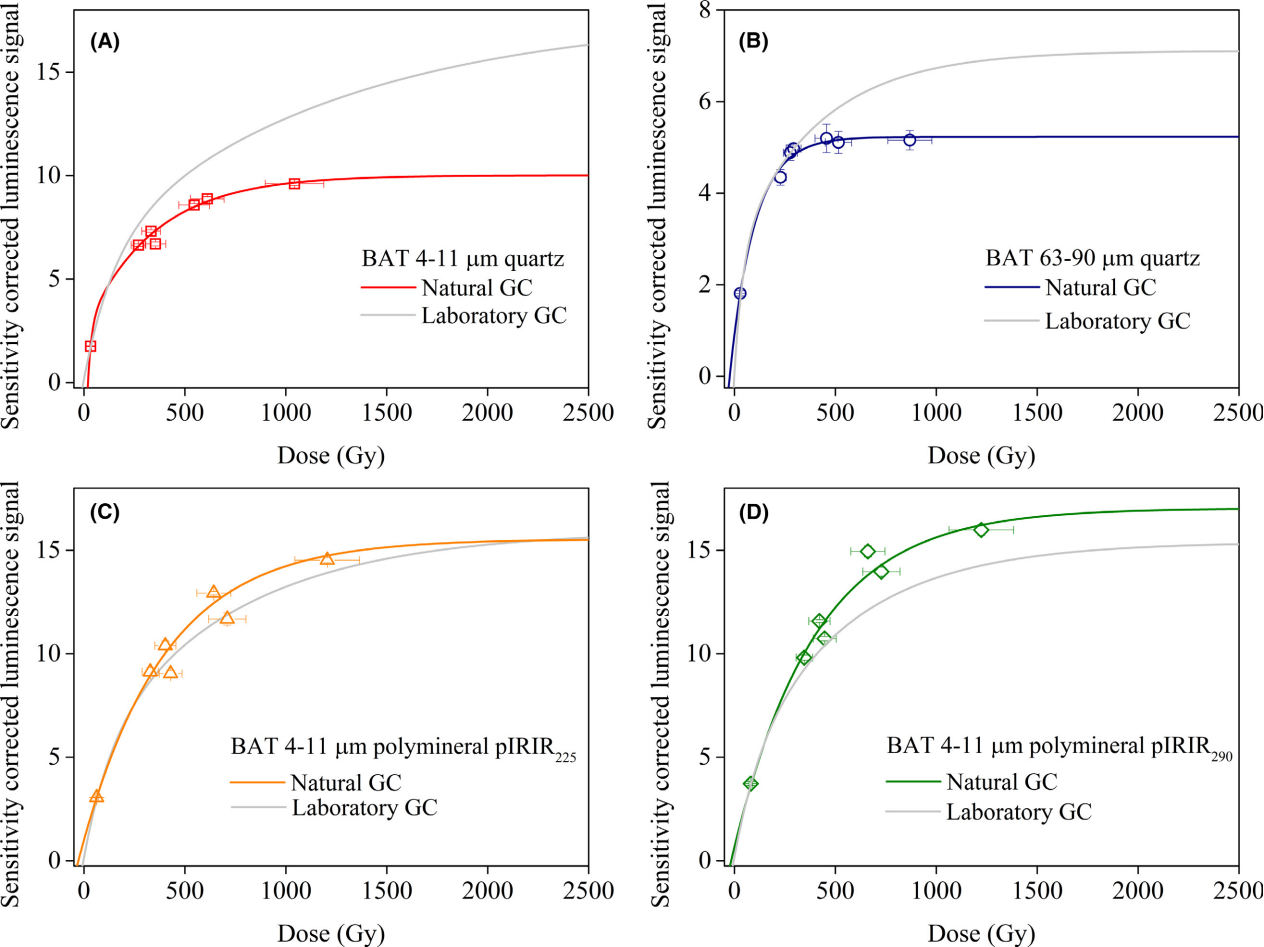
Average of natural sensitivity‐corrected luminescence signals emitted by (A) 4–11 μm and (B) 63–90 μm quartz, 4–11 μm polymineral grains measured with (C) pIRIR_225_ and (D) pIRIR_290_ protocols, plotted as function of expected equivalent doses for the samples collected from the loess–palaeosol boundaries (BAT‐1.0, BAT‐1.11, BAT‐1.12A, BAT‐1.12B, BAT‐1.16, BAT‐1.17, BAT‐1.19A). The expected equivalent doses are derived from the magnetic susceptibility data correlated to the benthic oxygen stack (Lisiecki & Raymo 2005). The average laboratory dose response is presented for comparison. Data are fitted by the sum of two saturating exponential functions. Note the small vertical uncertainties on the corrected natural light levels.

To construct natural dose response curves, the average sensitivity‐corrected natural luminescence signals (L_nat_/T_nat_) of the samples listed in Table S2 were plotted against the expected equivalent doses determined above. The natural growth curves are further compared to average laboratory dose response curves in order to assess the reliability of results (Fig. 8). Fine grained quartz natural and laboratory growth curves overlap until ~150 Gy (Fig. 8A). This suggests that fine quartz natural signals resulting from doses up to ~150 Gy (*c. *50 ka) could result in reliable ages. For higher natural doses the SAR protocol underestimates the expected fine quartz equivalent doses. Over the entire dose range investigated here, neither the natural nor the laboratory dose response curves reach full saturation. This may be due to the dose range being lower than 1200 Gy. This indicates that the fine quartz signals are outside field and laboratory saturation for the entire dose range investigated (Figs S7, 7A).

Timar‐Gabor & Wintle (2013) reported similar results for fine quartz extracted from the Costinesti LPS in Romania. The natural and laboratory dose response curves overlap up to ~200 Gy.

**Figure 9:**
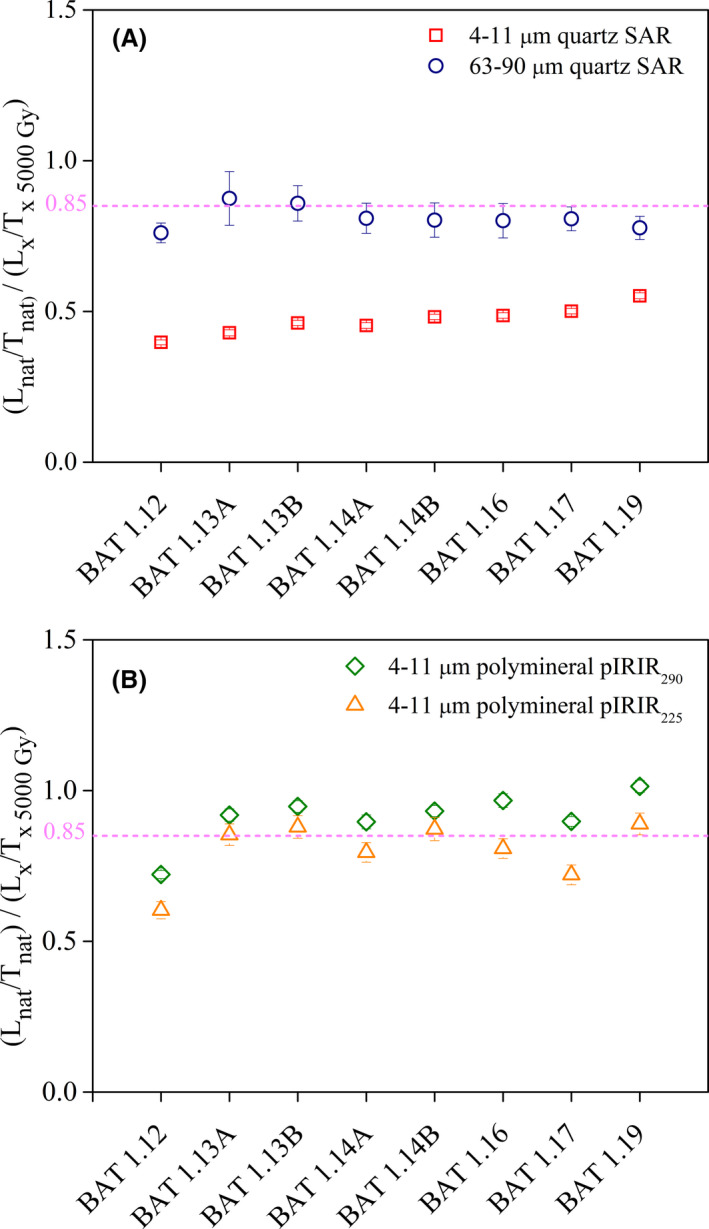
Ratios of the sensitivity‐corrected natural signals to the sensitivity‐corrected luminescence signals induced by a 5000 Gy dose calculated for (A) quartz and (B) polymineral fine grain samples collected from below the Last Interglacial palaeosol. The threshold of saturation is given by dashed lines and it is set at 0.85. Please note that the laboratory signal emitted by fine quartz continues to grow to doses higher than 5000 Gy (Fig. 7A).

Coarse‐grained quartz natural and laboratory growth curves overlap until ~250 Gy, corresponding to an upper age limit of *c. *100 ka for the Batajnica samples (Fig. 8B). This implies that for natural doses up to 250 Gy (*c. *100 ka), the SAR protocol provides reliable coarse quartz OSL ages. Figs 9A, S7 show that the natural signals emitted by coarse quartz samples with measured equivalent doses higher than ~250 Gy (BAT‐1.13A to 1.19A) reach field saturation but underestimate the full laboratory saturation by roughly 15%. Similar results have been reported for ‘infinitely old’ loess samples from Mircea‐Voda LPS in Romania (Timar‐Gabor *et al*. 2012) where the laboratory signals overestimate the natural field saturation by 10–15%.

The results obtained on 4–11 and 63–90 μm quartz from Batajnica confirm also the findings of Chapot *et al*. (2012) for 35–63 μm quartz extracted from Chinese loess. They recommend a maximum limit for OSL dating for an equivalent dose of 150 Gy after observing the overlap between the natural and laboratory dose response curves.

The uncorrected pIRIR_225_ natural and laboratory growth curves appear to generally overlap over the dose range investigated (up to ~1200 Gy; Fig. 8C). However, the overlap beyond ~500 Gy should be interpreted with caution because of the large errors of the data points and their spread along with our observation that the natural signals are in field saturation (Fig. S7).

The natural pIRIR_290_ signals are consistent with the laboratory signals for doses up to ~400 Gy (Fig. 8D). For higher doses, the natural pIRIR_290_ signals overestimate the laboratory signals for the Batajnica samples. Figs 9B, S7 show that the pIRIR_225_ and pIRIR_290_ natural signals are in both field and laboratory saturation for samples collected from L2 loess, starting with BAT‐1.13A. Similar results have been reported for the pIRIR_290_ protocol by Murray *et al*. (2014) on Stari Slankamen LPS, Serbia.

### Regional chronostratigraphical correlations

Loess records in the southern Middle Danube basin have been investigated intensively for palaeoclimate reconstructions (e.g. Marković *et al*. 2006, 2007, 2014, 2015; Bokhorst *et al*. 2009; Buggle *et al*. 2009; Stevens *et al*. 2011; Obreht *et al*. 2019). In particular, trends observed in environmental magnetic proxies have allowed for insights into the regional past long‐term climatic and environmental variability, as well as attempts at chronostratigraphical correlations amongst different sections (Marković *et al*. 2015). The magnetic susceptibility records from Batajnica (Marković *et al*. 2009), Stari Slankamen (Marković *et al*. 2011), Rogulić gully and other sections at the Titel loess plateau (Bokhorst *et al*. 2009; Basarin *et al*. 2014; Perić *et al*. 2019), Surduk (Antoine *et al*. 2009) and Crvenka (Stevens *et al*. 2011; Marković *et al*. 2018b) display similar variability for LGC (Fig. 2). Nevertheless, despite the available luminescence data for Irig (Marković *et al*. 2007), Surduk (Fuchs *et al*. 2008), Stari Slankamen (Schmidt *et al*. 2010; Murray *et al*. 2014), Titel (Perić *et al*. 2019), Crvenka (Stevens *et al*. 2011) and Orlovat (Timar‐Gabor *et al*. 2015), the coherence in palaeoclimate reconstructions amongst different LPS from the southern Middle Danube basin has only been evaluated by astronomical tuning (Basarin *et al*. 2014) and has not been discussed and tested in the framework of absolute luminescence dating.

By comparing the existing loess chronologies for individual sites in Serbia with available magnetic susceptibility records (this study and Antoine *et al*. 2009; Buggle *et al*. 2009; Schmidt *et al*. 2010; Stevens *et al*. 2011; Perić *et al*. 2019; Orlovat is not considered due to very strong influence of local conditions (Obreht *et al*. 2015)), it can be stated that similarities in magnetic susceptibility variations are also chronologically coherent. Below the Holocene S0 soil, intervals with low magnetic susceptibility values are consistently dated to between 20–35 ka. In particular, this period is characterized by similarly high dust accumulation rates in the whole region. As an illustrative example, samples at ~2 m depth in all sections with comparable sedimentation rates (Batajnica, Stari Slankamen, Titel and Crvenka) show an age range between 20–30 ka, while samples at ~4.5 m are consistently dated to *c. *35 ka (Schmidt *et al*. 2010; Stevens *et al*. 2011; Perić *et al*. 2019, and this study).

Beyond this interval (that can be tentatively related to MIS 2), all existing records in the southern Carpathian Basin are dated at considerably lower resolution (with the exception of the Titel LPS dated by Perić *et al*. (2019) but with fine quartz ages older than 35 ka considered unreliable), and comparison is more challenging. However, it is evident that the interval 35–40 ka reflects increased variability in magnetic susceptibility values pointing to pedogenesis phases that resulted in weak palaeosol formation (i.e. the L1SS1 according to Marković *et al*. 2008, 2015). The following interval is characterized by a decrease in magnetic susceptibility observed at all compared sections, but dated only at the Batajnica (this study) and Crvenka (Stevens *et al*. 2011) sections at *c. *50 ka. Stevens *et al*. (2011) linked this interval to the strong local influence of Heinrich Event 4. Although this needs further confirmation, it could also reflect to some degree the impact of the Campanian Ignimbrite/Y‐5 ash fall, similarly to what has been observed in the Lower Danube loess records (Veres *et al*. 2013; Obreht *et al*. 2017; Zeeden *et al*. 2018b). The next interval comprising loess and very weakly expressed palaeosols is characterized by another increase in magnetic susceptibility that probably represents the early MIS 3. However, no direct age estimates are available for this interval and we cannot provide further chronological constraints. Between this layer and the S1 palaeosol, a typical loess layer (L1LL2) with low magnetic susceptibility has been tentatively related to MIS 4 by pedostratigraphical correlative techniques (Marković *et al*. 2015). For the upper boundary, luminescence ages from Batajnica place L1LL2 between *c. *50 and 58 ka, thus rather within late MIS 4. The L1/S1 transition is placed around 74 and 84 ka based on coarse quartz and pIRIR_225_ ages. Interestingly, in almost all other sections with available age control over L1/S1 (Orlovat, Crvenka, Stari Slankamen), this transition is placed around 60–70 ka. This would suggest that the S1 palaeosol as developed in loess might encompass most of MIS 5 (Marković *et al*. 2008, 2009, 2015), and not only the Last Interglacial optimum (MIS 5e) as previously Bronger (2003) proposed. An exception is the Surduk section where the S1/L1 transition has been placed between 75–90 ka (Fuchs *et al*. 2008).

Furthermore, establishing secure chronological constraints beyond the LGC is very challenging considering limitations in numerical dating, as indicated in this study. However, a more secure regional correlation can be achieved using a tephrostratigraphical approach (Veres *et al*. 2013; Insinga *et al*. 2014; Leicher *et al*. 2016). In southeastern Europe, regional correlation has been widely applied relying on the L2 tephra recorded in many LPS (e.g. Vandenberghe *et al*. 2014; Marković *et al*. 2015, 2018a; Obreht *et al*. 2016). Samples BAT‐1.13A, B and BAT‐1.14A, B (Fig. 2) were collected in order to attempt constraining the depositional time of the two visible tephra layers identified within loess unit L2 (equivalent to MIS 6; Marković *et al*. 2015). Although glass shards were identified in both tephra layers (Fig. S8) they are altered and microprobe analyses did not allow for diagnostic geochemical fingerprinting. However, as seen in Fig. 2, the upper L2 tephra is clearly visible in the magnetic susceptibility data, and similar features have been reported in several MIS 6 loess records from the Adriatic coast to the Black Sea area (Laag *et al*. 2018). Based on a similar tephra report in Bulgaria, Antoine *et al*. (2019) proposed a correlation with Vico B‐ignimbrite dated at 162±8 ka but without reporting any verifiable geochemical data in support. Therefore, it shall be stated that the upper L2 tephra could potentially be the equivalent of one of the tephra layers found in Lake Ohrid (e.g. OH‐DP‐0617‐Vico B at 162±6 ka and OH‐DP‐0624‐CF‐V5‐Pitigliano Tuff at 163±22 ka; Leicher *et al*. 2016) and in the Fucino basin (e.g. TF‐15, TF‐16, TF‐17; Giaccio *et al*. 2017) and dated to 150–160 ka. The Mediterranean Sea tephra data also document several major tephra layers between 140–170 ka (Insinga *et al*. 2014). Moreover, as seen also at Batajnica, at least two tephra layers are preserved regionally within the L2 loess; this highlights the risk in deriving ages without proper geochemical control, especially when at the limit of reliable luminescence dating (Table 1).

## Conclusions

Luminescence dating of 63–90 μm quartz and 4–11 μm polymineral grains using the pIRIR_225_ protocol provided age estimates for the LGC at Batajnica that generally agree with the previously established correlative chronological scheme proposed by Marković *et al*. (2009). The 63–90 μm quartz ages are reliable up to 100 ka before exhibiting field and laboratory saturation. The 4–11 μm quartz yields reliable ages only for MIS 2 loess samples and increasingly underestimates the true depositional time for older samples. The pIRIR_225_ and pIRIR_290_ protocols applied on 4–11 μm polymineral grains provide reliable ages over the LGC, beyond which they are in field and laboratory saturation. Interpretation of the pIRIR_225_ and pIRIR_290_ ages obtained immediately below the Last Interglacial palaeosol is hampered by different results yielded by doublet samples.

The upper limit of the luminescence dating methods at Batajnica is imposed by saturation of the natural signals starting with samples collected from L2 loess (BAT‐1.13A‐1.19A, B).

The observed similarities in pacing of magnetic susceptibility records in the majority of sites in northern Serbia are chronologically coherent with the Batajnica section and match global records. This allowed us to construct natural dose response curves. The comparison between the natural and laboratory dose response curves predicts the dose range over which reliable luminescence ages are obtained for each signal investigated. For 4–11 and 63–90 μm quartz, the natural and laboratory dose response curves overlap until ~150 and ~250 Gy, respectively, while for pIRIR_225_ there is an apparent overlap of the natural growth curve up to at least 500 Gy. The pIRIR_290_ natural and laboratory growth curves are consistent with the laboratory dose response curve up to doses of 400 Gy beyond which the natural signals overestimate the laboratory signals.

## Author contributions

Conceptualization and funding acquisition: AT‐G; sample collection: DV; methodology: AA and DC; luminescence measurements: AA; alpha and gamma spectrometry measurements: SK; data analysis: DC and AA; magnetic susceptibility correlation: UH, IO and SBM; data presentation: AA, DC and IO; original draft preparation: DC, AA, DV, IO, UH, SBM; review and editing: all authors; supervision: AT‐G.

## Supporting information


*Fig. S1*. The ratio between the ^210^Pb and ^226^Ra (^214^Pb and ^214^Bi peaks) concentrations determined by gamma spectrometry.

*Fig. S2*. The ratio between ^210^Pb concentration determined by gamma spectrometry and ^210^Po concentration measured by alpha spectrometry.

*Fig. S3*. Representative sensitivity‐corrected dose response curves constructed for sample BAT‐1.10 using one aliquot of (A) fine (4–11 μm) quartz grains, (B) coarse (63–90 μm) quartz grains and (C, D) polymineral fine (4–11 μm) grains.

*Fig. S4*. Equivalent dose dependence on preheat temperatures for fine quartz fraction from sample BAT‐1.13B.

*Fig. S5*. Results of the fading rate measurements on individual aliquots of 4–11 μm polymineral material and 4–11 and 63–90 μm quartz.

*Fig. S6*. Luminescence ages of quartz and polymineral fine grains.

*Fig. S7*. Average values for the natural sensitivity‐corrected luminescence signals for all the investigated aliquots.

*Fig. S8*. Glass shards identified in the upper tephra layer (A) and lower tephra layer (B) in the L2 unit.

*Table S1*. Flowchart of the SAR‐OSL (Murray & Wintle 2000, 2003), pIRIR_290_ (Buylaert *et al*. 2011b, 2012; Thiel *et al*. 2011a) and pIRIR_225_ (Buylaert *et al*. 2009; Wacha & Frechen 2011; Vasiliniuc *et al*. 2012) protocols applied in this study.

*Table S2*. Information on the samples used for the construction of the natural dose response curves.

*Table S3*. Summary of dosimetry data.

*Table S4*. The measured equivalent doses along with the performance parameters of the SAR‐OSL protocol as well as for pIRIR_290_ and pIRIR_225_ for each quartz grain‐size and polymineral fine grains.

*Table S5*. The measured residual doses along with the performance parameters of the pIRIR_290_ procedure for fine (4–11 μm) polymineral grains of the 10 samples analysed.

*Table S6*. The measured residual doses along with the performance parameters of the pIRIR_225_ procedure for fine (4–11 μm) polymineral grains of the nine samples analysed.

*Table S7. g‐*values measured on polymineral fine grains using the pIRIR_290_ and pIRIR_225_ protocols as well as on fine and coarse quartz using the SAR‐OSL protocol.

*Table S8*. The effect of adding large doses on top of the naturally accrued dose for sample BAT‐1.19A using SAR‐OSL, pIRIR_225_ and pIRIR_290_ protocols.

*Table S9*. Maximum corrected luminescence signals in the SAR‐OSL and pIRIR protocols used and natural corrected luminescence signals for the sample BAT‐1.19A.

*Table S10*. Saturation characteristics for dose response curves up to 5000 Gy on BAT‐1.19A using different test doses.

*Table S11*. Natural corrected luminescence signals for the samples BAT‐1.12A–BAT‐1.19B and maximum corrected luminescence signals recorded in the SAR‐OSL and post‐IR IRSL protocols.

*Data S1*. Sample preparation for alpha spectrometry.
