# Supplementary material for: Testing polymineral post‐IR IRSL and quartz SAR‐OSL protocols on Middle to Late Pleistocene loess at Batajnica, Serbia
Source: Boreas. 2020 May 4;49(3):615–33. doi: 10.1111/bor.12442 (PMC7508060; doi:10.1111/bor.12442)
Supplement: Supplementary file 1 — Fig. S1. The ratio between the 210Pb and 226Ra (214Pb and 214Bi peaks) concentrations determined by gamma spectrometry. [file BOR-49-615-s001.docx]

**
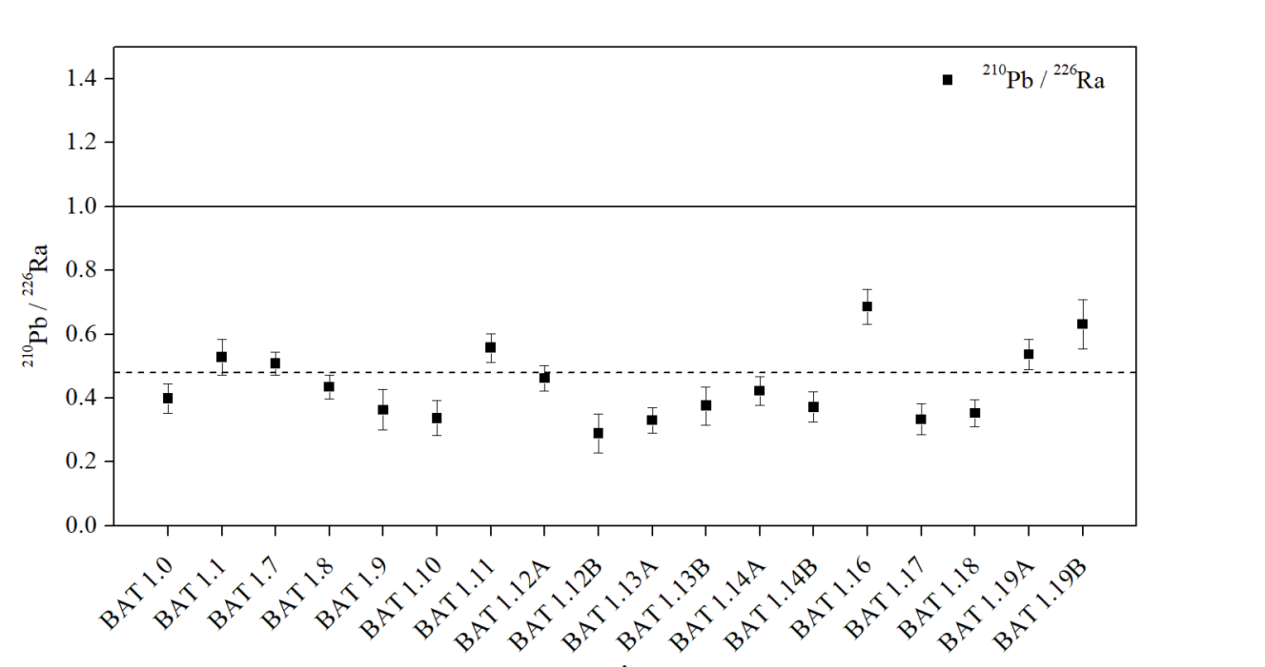
**

Figure S1. The ratio between the ^210^Pb and ^226^Ra (^214^Pb and ^214^Bi peaks) concentration determined by gamma spectrometry. The solid line indicates the ideal value while with dashed line is represented the average value of the ratios measured for all samples (0.48±0.03).
