# Supplementary material for: Testing polymineral post‐IR IRSL and quartz SAR‐OSL protocols on Middle to Late Pleistocene loess at Batajnica, Serbia
Source: Boreas. 2020 May 4;49(3):615–33. doi: 10.1111/bor.12442 (PMC7508060; doi:10.1111/bor.12442)
Supplement: Supplementary file 2 — Fig. S2. The ratio between 210Pb concentration determined by gamma spectrometry and 210Po concentration measured by alpha spectrometry. [file BOR-49-615-s002.docx]

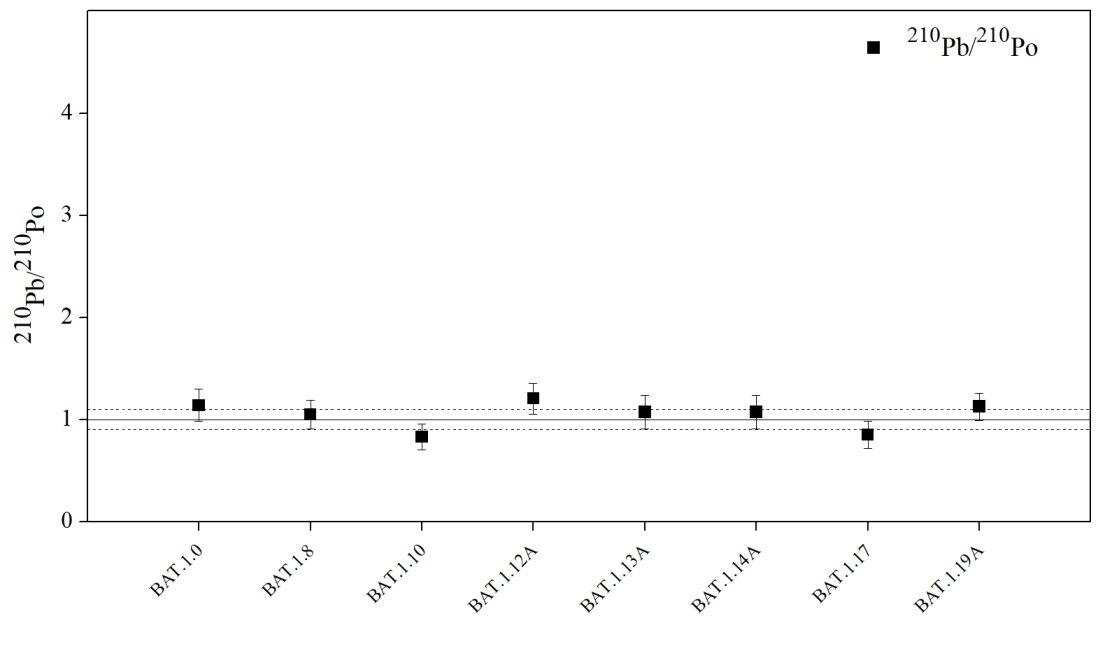


Figure S2. The ratio between ^210^Pb concentration determined by gamma spectrometry and ^210^Po measured by alpha spectrometry. The solid line represents the ideal value of the ratio whereas the dotted lines represent 10% deviation from unity.
