# Supplementary material for: Testing polymineral post‐IR IRSL and quartz SAR‐OSL protocols on Middle to Late Pleistocene loess at Batajnica, Serbia
Source: Boreas. 2020 May 4;49(3):615–33. doi: 10.1111/bor.12442 (PMC7508060; doi:10.1111/bor.12442)
Supplement: Supplementary file 3 — Fig. S3. Representative sensitivity‐corrected dose response curves constructed for sample BAT‐1.10 using one aliquot of (A) fine (4–11 μm) quartz grains, (B) coarse (63–90 μm) quartz grains and (C, D) polymineral fine (4–11 μm) grains. [file BOR-49-615-s003.docx]

| 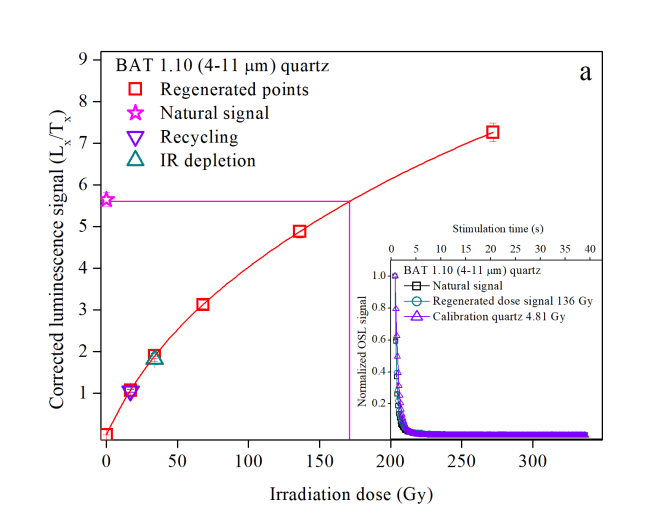 | 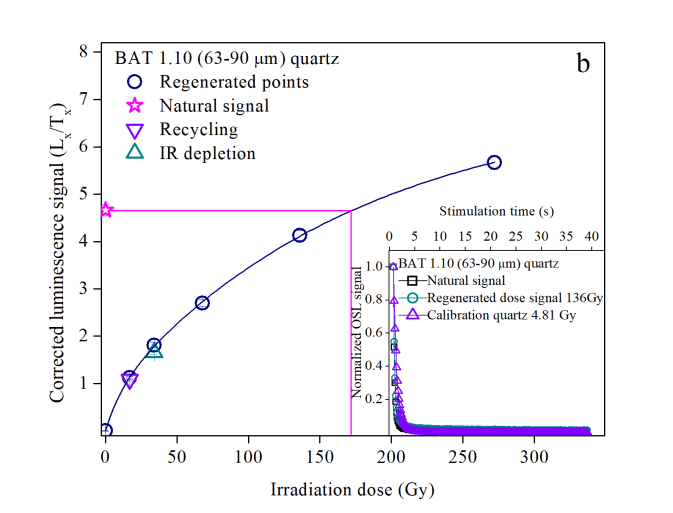 |
| --- | --- |
| 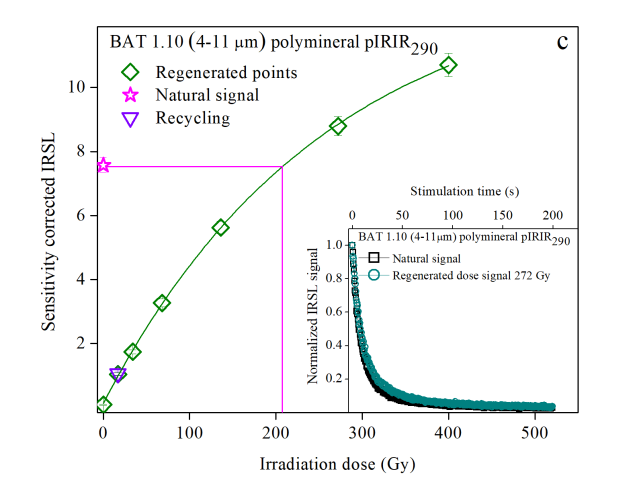 | 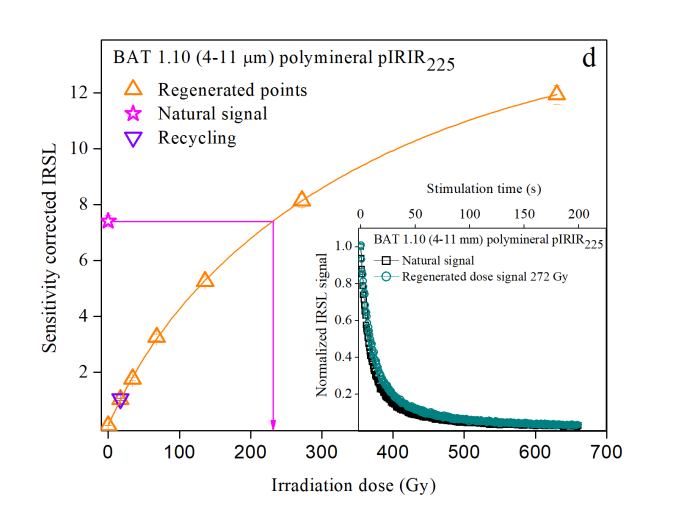 |

Figure S3. Representative sensitivity-corrected dose response curves constructed for sample BAT-1.10 using one aliquot of (A) fine (4-11 µm) quartz grains, (B) coarse (63-90 µm) quartz grains and (C) (D) polymineral fine (4-11 µm)grains. The sensitivity corrected natural signals is depicted as a star and a line indicates the equivalent dose. Recycling and IR depletion points are represented as an inverse triangle and up triangle, respectively. The inset show a typical decay curve of natural CW-OSL signal (open squares) in comparison to a regenerated signal (open circles) induced by a beta dose approximately equal with the equivalent dose and a calibration quartz signal (open triangles) in the case of quartz data, while for pIRIR_290_ and pIRIR_225_ data, natural signals are compared to regenerative signals.
