# Supplementary material for: Testing polymineral post‐IR IRSL and quartz SAR‐OSL protocols on Middle to Late Pleistocene loess at Batajnica, Serbia
Source: Boreas. 2020 May 4;49(3):615–33. doi: 10.1111/bor.12442 (PMC7508060; doi:10.1111/bor.12442)
Supplement: Supplementary file 4 — Fig. S4. Equivalent dose dependence on preheat temperatures for fine quartz fraction from sample BAT‐1.13B. [file BOR-49-615-s004.docx]

Figure S4. Equivalent dose dependence on preheat temperatures for fine quartz fraction from sample BAT-1.13B. A cutheat (test dose preheat) of 180 ̊ C was employed
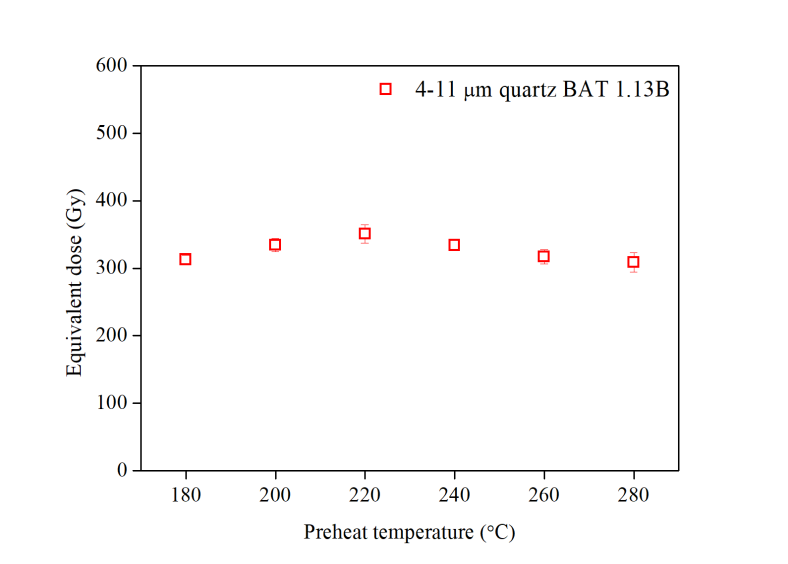
.
