# Supplementary material for: Testing polymineral post‐IR IRSL and quartz SAR‐OSL protocols on Middle to Late Pleistocene loess at Batajnica, Serbia
Source: Boreas. 2020 May 4;49(3):615–33. doi: 10.1111/bor.12442 (PMC7508060; doi:10.1111/bor.12442)
Supplement: Supplementary file 5 — Fig. S5. Results of the fading rate measurements on individual aliquots of 4–11 μm polymineral material and 4–11 and 63–90 μm quartz. [file BOR-49-615-s005.docx]

| A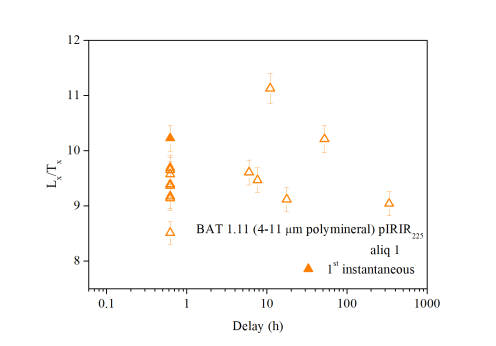 | B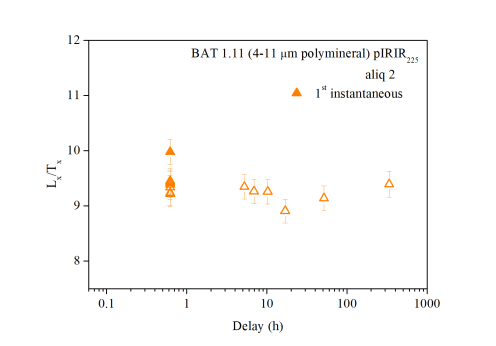 | C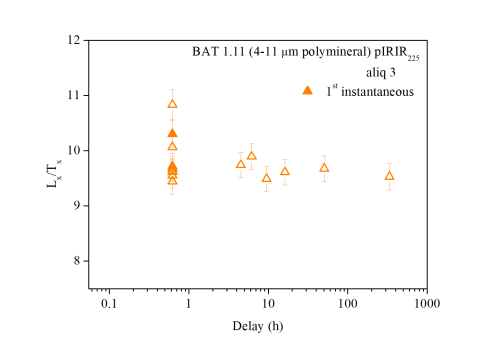 | D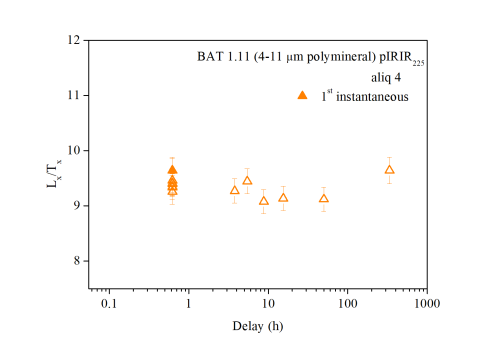 |
| --- | --- | --- | --- |
| E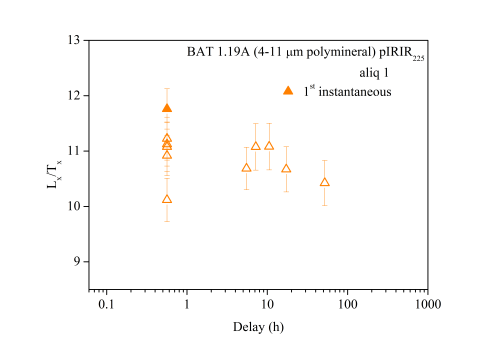 | F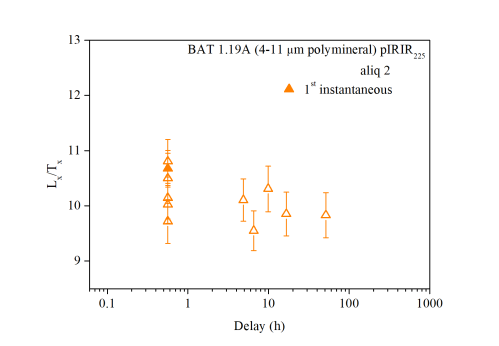 | G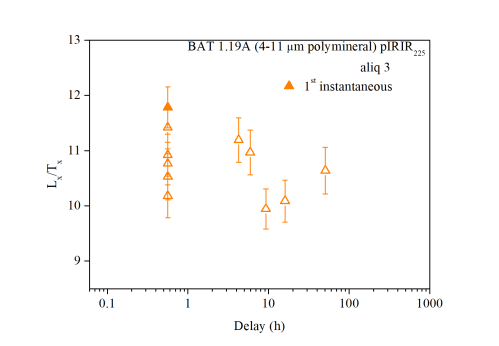 | H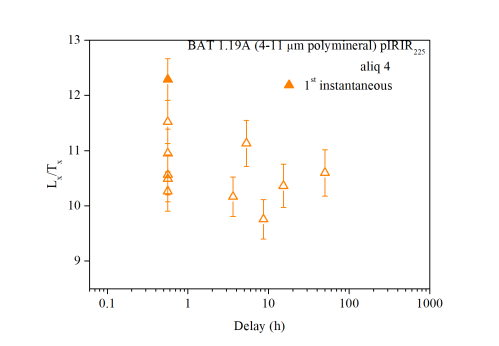 |
| I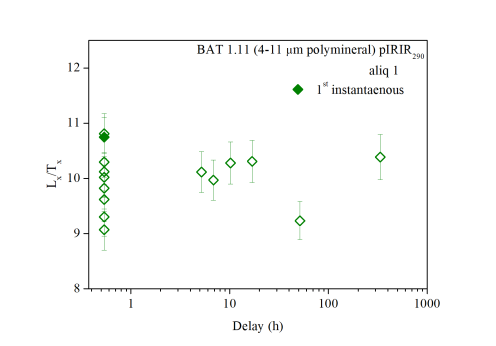 | J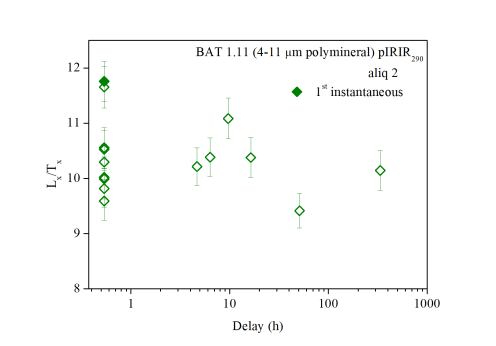 | K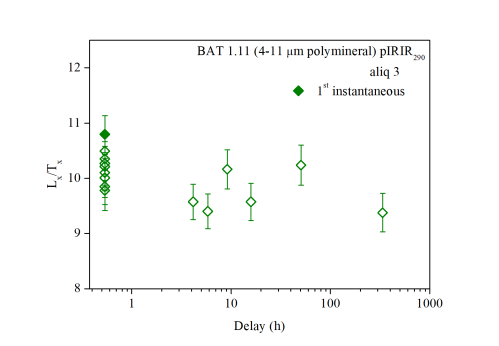 | L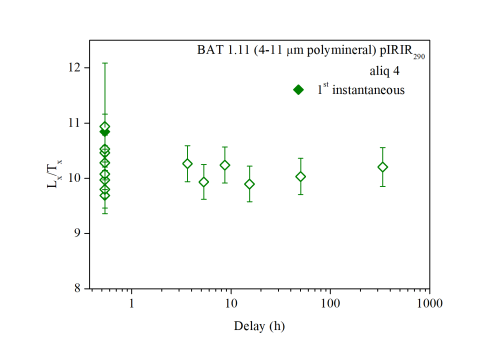 |

| M | N | O | P |
| --- | --- | --- | --- |
| Q | R | S |  |

Figure S5. Results of the fading rate measurements on individual aliquots of 4-11 µm polymineral material, 4-11 µm quartz and 63-90 µm quartz. (A-D) pIRIR_225_ protocol on sample BAT-1.11, (E-H) pIRIR_225_ protocol on sample BAT-1.19A, (I-L) pIRIR_290_ protocol on sample BAT-1.11, (M-P)SAR-OSL protocol on sample BAT-1.11 using coarse quartz and (Q-S) SAR-OSL protocol on sample BAT-1.11 using fine quartz. The signals were read after a maximum delay of 14 days for sample BAT-1.11 while for sample BAT-1.19A the maximum delay was 2 days. The highest storage period was 21 days in the case of 4-11 µm quartz and 23 days for 63-90 µm quartz. A number of 4 consecutive prompt read-outs were carried out before signal measurement after delay and two consecutive prompts read-outs were added after different delays times. The signal intensity of the first instantaneous read-out is represented with filled symbols.
