# Supplementary material for: Testing polymineral post‐IR IRSL and quartz SAR‐OSL protocols on Middle to Late Pleistocene loess at Batajnica, Serbia
Source: Boreas. 2020 May 4;49(3):615–33. doi: 10.1111/bor.12442 (PMC7508060; doi:10.1111/bor.12442)
Supplement: Supplementary file 6 — Fig. S6. Luminescence ages of quartz and polymineral fine grains. [file BOR-49-615-s006.docx]

| 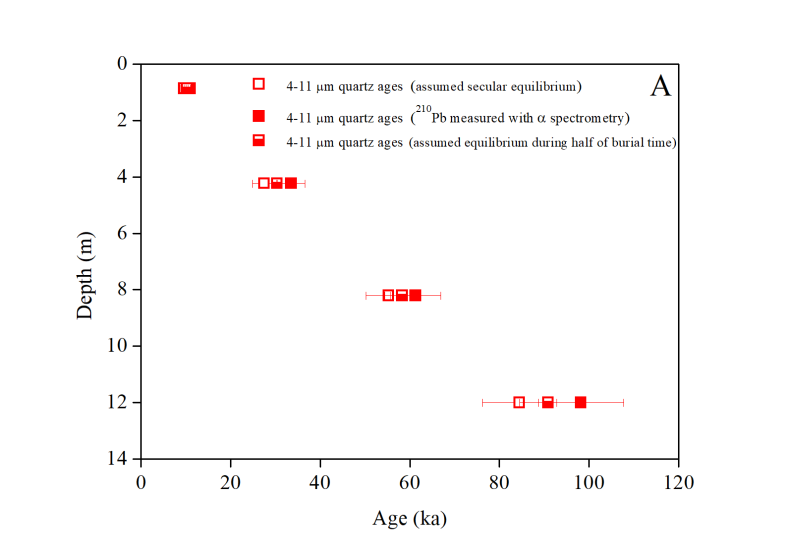 | 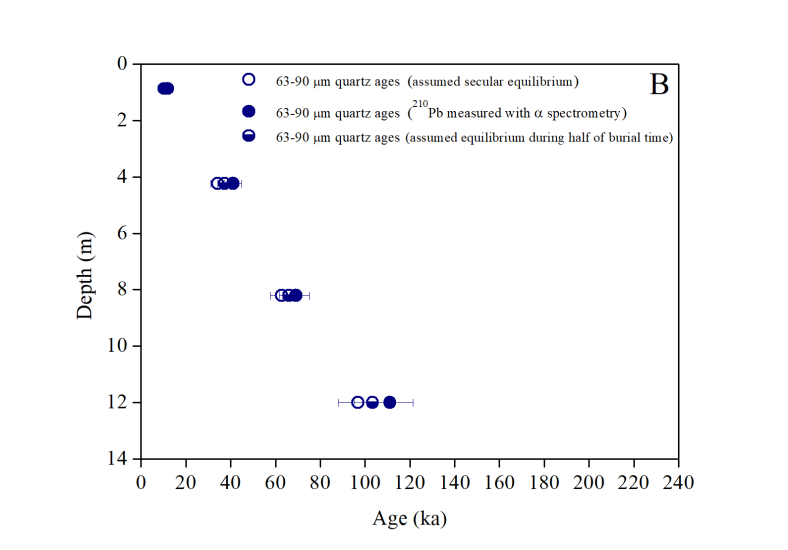 |
| --- | --- |
|  |  |

Figure S6. Luminescence ages on quartz and polymineral fine grains. Ages obtained by assuming secular equilibrium between ^222^Ra and ^210^Pb are presented with open symbols while ages calculated using the measured concentration of ^238^U (^234^Th: 92.3keV and 92.8 keV peaks) and ^222^Ra (^214^Pb: 351 keV and 295 keV peaks; ^214^Bi: 609 keV peak) based on gamma spectrometry, and the concentration of ^210^Pb determined by alpha spectrometry are represented with filled symbols, assuming present day degree of radioactive disequilibrium. The OSL ages given with half coloured symbols were calculated assuming radioactive equilibrium for half of the burial time while for the other half the concentration of ^210^Pb was taken into account.
