# Supplementary material for: Testing polymineral post‐IR IRSL and quartz SAR‐OSL protocols on Middle to Late Pleistocene loess at Batajnica, Serbia
Source: Boreas. 2020 May 4;49(3):615–33. doi: 10.1111/bor.12442 (PMC7508060; doi:10.1111/bor.12442)
Supplement: Supplementary file 7 — Fig. S7. Average values for the natural sensitivity‐corrected luminescence signals for all the investigated aliquots. [file BOR-49-615-s007.docx]

| 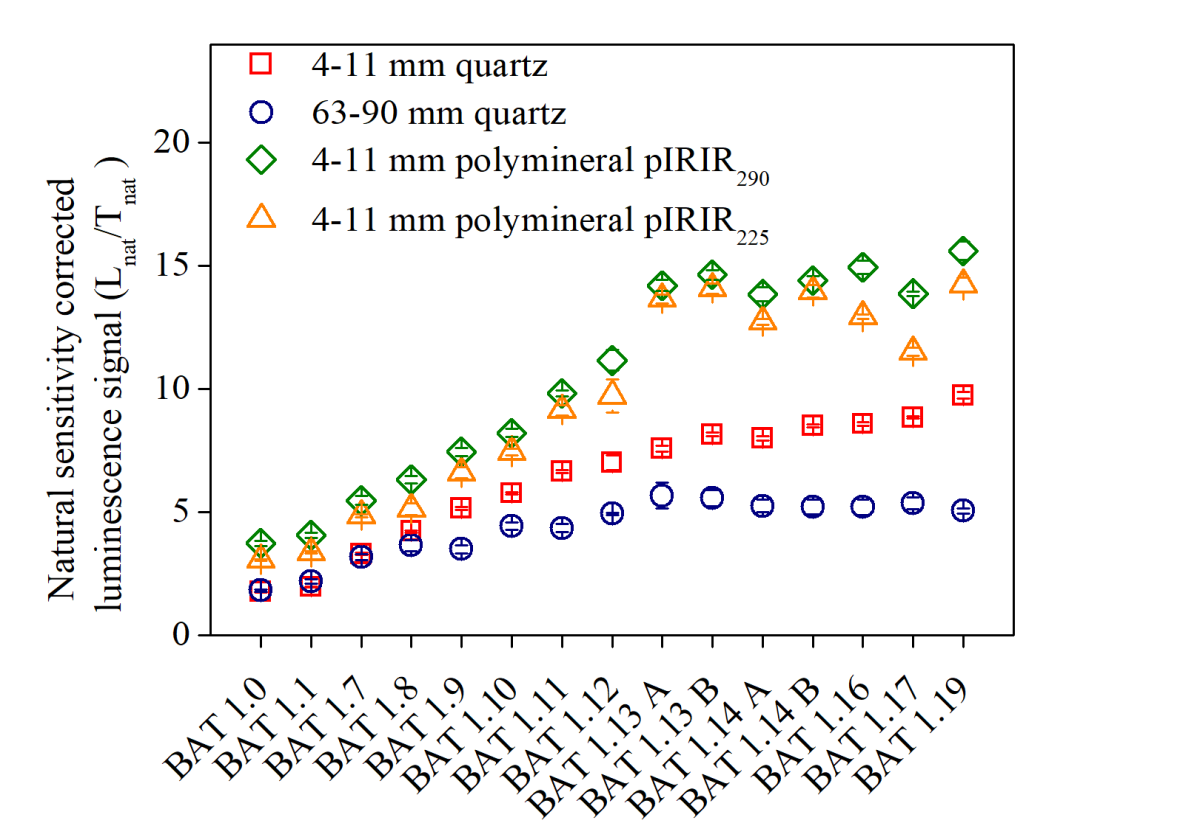 |
| --- |
| Figure S7. Average values for the natural sensitivity corrected luminescence signals for all the investigated aliquots. Data for BAT-1.12, BAT-1.17 and BAT-1.19 represent the average of the doublet samples. |
