# Supplementary figures and images for: Testing polymineral post‐IR IRSL and quartz SAR‐OSL protocols on Middle to Late Pleistocene loess at Batajnica, Serbia
Source: Boreas. 2020 May 4;49(3):615–33. doi: 10.1111/bor.12442 (PMC7508060; doi:10.1111/bor.12442)

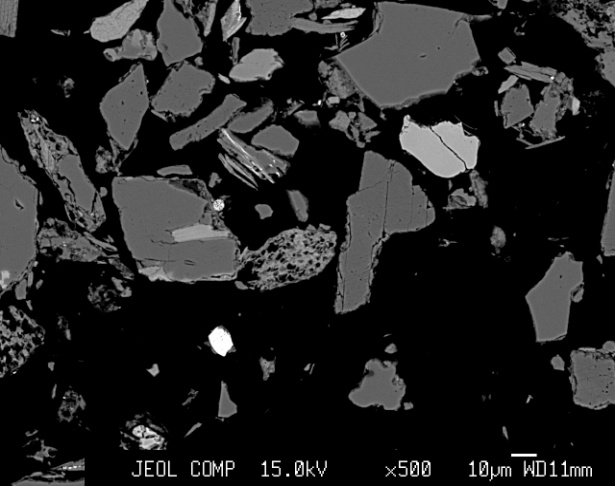

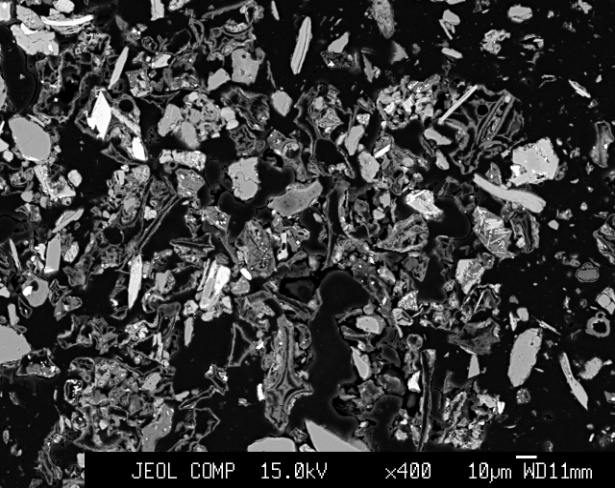
A) B)

Figure S8. Glass shards identified in the A) upper tephra layer and B) lower tephra layer in L2 unit.

Supplement: Supplementary file 8 — Fig. S8. Glass shards identified in the upper tephra layer (A) and lower tephra layer (B) in the L2 unit. [file BOR-49-615-s008.docx]
