# Supplementary material for: Testing polymineral post‐IR IRSL and quartz SAR‐OSL protocols on Middle to Late Pleistocene loess at Batajnica, Serbia
Source: Boreas. 2020 May 4;49(3):615–33. doi: 10.1111/bor.12442 (PMC7508060; doi:10.1111/bor.12442)
Supplement: Supplementary file 9 — Table S1. Flowchart of the SAR‐OSL (Murray & Wintle 2000, 2003), pIRIR290 (Buylaert et al. 2011b, 2012; Thiel et al. 2011a) and pIRIR225 (Buylaert et al. 2009; Wacha & Frechen 2011; Vasiliniuc et al. 2012) protocols applied in this study. [file BOR-49-615-s009.docx]

Table S1. Flowchart of the SAR-OSL on quartz (Murray & Wintle 2000, 2003), pIRIR_290_ (Thiel *et al.* 2011a; Buylaert *et al.* 2011b, 2012) and pIRIR_225_ (Buylaert *et al.* 2009; Wacha & Frechen 2011; Vasiliniuc *et al*. 2012) protocols applied in this study.(*) Unless otherwise stated.

| step | a. SAR protocol | b. pIRIR_290_ | c. pIRIR_225_ |
| --- | --- | --- | --- |
| 1 | Dose | Dose | Dose |
| 2 | Preheat (125 ̊ C; 10s) | Preheat (320 ̊ C; 60s) | Preheat (250 ̊ C; 60s) |
| 3 | Blue OSL (125 ̊ C; 40s) | IRSL (50 ̊ C; 200s) | IRSL (50 ̊ C; 200s) |
| 4 | Test dose (17Gy)* | IRSL (290 ̊ C; 200s) | IRSL (225 ̊ C; 200s) |
| 5 | Cutheat (180 ̊ C) | Test dose (17Gy)* | Test dose (17Gy)* |
| 6 | Blue OSL (125 ̊ C; 40s) | Preheat (320 ̊ C; 60s) | Preheat (250 ̊ C; 60s) |
| 7 | Blue OSL (280 ̊ C; 40s) | IRSL (50 ̊ C; 200s) | IRSL (50 ̊ C; 200s) |
| 8 |  | IRSL (290 ̊ C; 200s) | IRSL (225 ̊ C; 200s) |
| 9 |  | IRSL (325 ̊ C; 100s) | IRSL (290 ̊ C; 100s) |
