# Supplementary material for: Testing polymineral post‐IR IRSL and quartz SAR‐OSL protocols on Middle to Late Pleistocene loess at Batajnica, Serbia
Source: Boreas. 2020 May 4;49(3):615–33. doi: 10.1111/bor.12442 (PMC7508060; doi:10.1111/bor.12442)
Supplement: Supplementary file 10 — Table S2. Information on the samples used for the construction of the natural dose response curves. [file BOR-49-615-s010.docx]

Table S2. Information on the samples used for the construction of the natural dose response curves. The expected ages are from correlation of the paleosol/loess boundaries with benthic isotope stack (Lisiecki & Raymo 2005). The expected equivalent dose (De) for each sample was calculated by multiplying the annual dose rate by the expected age.

| Sample code | Sampling information | Boundary age (ka) | Grain size (µm) | Annual dose (Gy/ka) | Expected De (Gy) |
| --- | --- | --- | --- | --- | --- |
| BAT 1.0 | L1/S0 | 11.5±1 | 4-11 quartz | 2.9±0.3 | 33±4 |
|  |  |  | 63-90 quartz | 2.4±0.2 | 28±3 |
|  |  |  | pIRIR_290_ | 3.2±0.3 | 37±5 |
|  |  |  | pIRIR_225_ | 3.2±0.3 | 37±5 |
| BAT 1.11 | S1/L1 | 80±8 | 4-11 quartz | 3.4±0.3 | 270±37 |
|  |  |  | 63-90 quartz | 2.9±0.2 | 228±28 |
|  |  |  | pIRIR_290_ | 3.8±0.3 | 302±40 |
|  |  |  | pIRIR_225_ | 3.8±0.3 | 302±40 |
| BAT 1.12A | L2/S1 | 130±13 | 4-11 quartz | 2.6±0.3 | 332±46 |
|  |  |  | 63-90 quartz | 2.1±0.2 | 277±35 |
|  |  |  | pIRIR_290_ | 2.9±0.3 | 377±52 |
|  |  |  | pIRIR_225_ | 2.9±0.3 | 377±52 |
| BAT 1.12B | L2/S1 | 130±13 | 4-11 quartz | 2.7±0.3 | 354±51 |
|  |  |  | 63-90 quartz | 2.3±0.2 | 293±37 |
|  |  |  | pIRIR_290_ | 3.1±0.3 | 403±56 |
|  |  |  | pIRIR_225_ | 3.1±0.3 | 403±56 |
| BAT 1.16 | S2/L2 | 191±19 | 4-11 quartz | 2.9±0.3 | 546±76 |
|  |  |  | 63-90 quartz | 2.4±0.2 | 456±57 |
|  |  |  | pIRIR_290_ | 3.2±0.3 | 617±84 |
|  |  |  | pIRIR_225_ | 3.2±0.3 | 617±84 |
| BAT 1.17 | L3/S2 | 228±23 | 4-11 quartz | 2.7±0.2 | 611±83 |
|  |  |  | 63-90 quartz | 2.3±0.2 | 515±65 |
|  |  |  | pIRIR_290_ | 3.0±0.3 | 684±92 |
|  |  |  | pIRIR_225_ | 3.0±0.3 | 684±92 |
| BAT 1.19A | Lower boundary S3 | 322±32 | 4-11 quartz | 3.2±0.3 | 1043±145 |
|  |  |  | 63-90 quartz | 2.7±0.2 | 869±109 |
|  |  |  | pIRIR_290_ | 3.7±0.3 | 1179±160 |
|  |  |  | pIRIR_225_ | 3.7±0.3 | 1179±160 |
