# Supplementary material for: Testing polymineral post‐IR IRSL and quartz SAR‐OSL protocols on Middle to Late Pleistocene loess at Batajnica, Serbia
Source: Boreas. 2020 May 4;49(3):615–33. doi: 10.1111/bor.12442 (PMC7508060; doi:10.1111/bor.12442)
Supplement: Supplementary file 11 — Table S3. Summary of dosimetry data. [file BOR-49-615-s011.docx]

Table S3. Summary of dosimetry data. The uncertainties associated with the annual doses are random. Specific activities were measured on well detector by gamma spectrometry and the ages were determined considering 15% water content; beta attenuation and etching factors used for 63-90 µm quartz were 0.94±0.05 (Mejdahl 1979); adopted alpha efficiency factor was 0.04±0.02 for 4-11 µm quartz and 0.08±0.02 for polymineral 4-11 µm fine grains, respectively (Rees-Jones 1995). The total dose rate consists of the contribution from the beta and gamma radiations for coarse grains as well as the contribution from alpha radiations in the case of fine grains. The contribution of cosmic radiation was taken into account and calculated accordingly to Prescott & Hutton (1994). For coarse quartz grains an internal dose rate of 0.01±0.002 Gy/ka was considered (Vandenberghe *et al.* 2008).

| **Sample code** | **Radionuclide activities** | | | | **Annual doses** | | | |
| --- | --- | --- | --- | --- | --- | --- | --- | --- |
|  | **^210^Pb** | **^232^Th** | **^226^Ra** | **^40^K** | **4-11 µm** | **63-90 µm** | **pIRIR_290_** | **pIRIR_225_** |
| **BAT 1.0** | 16±4 | 41.3±1.2 | 40.4±0.2 | 422±16 | 2.9±0.08 | 2.4±0.07 | 3.2±0.09 | 3.2±0.09 |
| **BAT 1.1** | 21±4 | 36.9±1.5 | 38.8±1.7 | 440±16 | 2.9±0.09 | 2.4±0.07 | 3.3±0.11 | 3.3±0.11 |
| **BAT 1.7** | 24±4 | 39.9±0.3 | 47.8±0.4 | 463±14 | 3.1±0.07 | 2.6±0.06 | 3.6±0.08 | 3.6±0.08 |
| **BAT 1.8** | 27±4 | 49.7±0.3 | 53.8±1.5 | 510±17 | 3.5±0.08 | 2.9±0.07 | 4.0±0.10 | 4.0±0.10 |
| **BAT 1.9** | 15±5 | 41.0±3.2 | 40.5±1.1 | 468±19 | 2.9±0.12 | 2.4±0.10 | 3.2±0.12 | 3.2±0.12 |
| **BAT 1.10** | 14±4 | 38.1±0.7 | 35.9±1.1 | 497±17 | 2.8±0.08 | 2.4±0.07 | 3.1±0.10 | 3.1±0.10 |
| **BAT 1.11** | 24±4 | 43.5±0.4 | 43.6±0.5 | 568±19 | 3.4±0.09 | 2.9±0.07 | 3.8±0.10 | 3.8±0.10 |
| **BAT 1.12A** | 22±4 | 36.5±0.4 | 38.1±1.2 | 373±14 | 2.6±0.08 | 2.1±0.06 | 2.9±0.09 | 2.9±0.09 |
| **BAT 1.12B** | 30±6 | 38.0±0.2 | 38.7±0.6 | 370±17 | 2.7±0.10 | 2.3±0.08 | 3.1±0.12 | 3.1±0.12 |
| **BAT 1.13A** | 15±4 | 35.7±1.7 | 35.5±0.4 | 418±15 | 2.5±0.09 | 2.1±0.07 | 2.9±0.09 | 2.9±0.09 |
| **BAT 1.13B** | 13±4 | 35.4±0.6 | 35.4±0.6 | 467±17 | 2.7±0.09 | 2.3±0.07 | 3.0±0.10 | 3.0±0.10 |
| **BAT 1.14A** | 16±4 | 37.7±0.6 | 38.8±0.6 | 409±16 | 2.6±0.08 | 2.2±0.06 | 2.9±0.09 | 2.9±0.09 |
| **BAT 1.14B** | 14±4 | 42.4±1.7 | 38.4±0.9 | 415±17 | 2.7±0.09 | 2.2±0.07 | 3.0±0.09 | 3.0±0.09 |
| **BAT 1.16** | 25±4 | 39.7±0.7 | 36.9±0.7 | 441±17 | 2.9±0.09 | 2.4±0.07 | 3.2±0.10 | 3.2±0.10 |
| **BAT 1.17** | 12±4 | 38.9±0.4 | 36.6±1.2 | 463±14 | 2.7±0.07 | 2.3±0.06 | 3.0±0.09 | 3.0±0.09 |
| **BAT 1.18** | 13±3 | 38.4±0.6 | 36.3±0.7 | 459±13 | 2.7±0.06 | 2.3±0.05 | 3.0±0.08 | 3.0±0.08 |
| **BAT 1.19A** | 20±4 | 54.6±1.5 | 38.0±1.5 | 496±15 | 3.2±0.08 | 2.7±0.07 |  | 3.4±0.09 |
| **BAT 1.19B** | 20±5 | 50.2±0.8 | 32.0±0.7 | 496±17 | 3.1±0.10 | 2.6±0.08 |  | 3.2±0.11 |
