# Supplementary material for: Testing polymineral post‐IR IRSL and quartz SAR‐OSL protocols on Middle to Late Pleistocene loess at Batajnica, Serbia
Source: Boreas. 2020 May 4;49(3):615–33. doi: 10.1111/bor.12442 (PMC7508060; doi:10.1111/bor.12442)
Supplement: Supplementary file 12 — Table S4. The measured equivalent doses along with the performance parameters of the SAR‐OSL protocol as well as for pIRIR290 and pIRIR225 for each quartz grain‐size and polymineral fine grains. [file BOR-49-615-s012.docx]

Table S4. The measured equivalent doses along with the performance parameters of the SAR-OSL protocol as well as for pIRIR_290_ and pIRIR_225_ for each quartz grain-size and polymineral fine grains. The number of aliquots (*n*) averaged for equivalent dose calculation is also given. Quoted errors represent 1σ. The equivalent doses given in italics are calculated by interpolating natural signals onto the saturation region of the dose response curve.

| Sample code | Depth | ED (Gy) | | | | Recycling | | | | IR depletion | | Recuperation (%) | | | |
| --- | --- | --- | --- | --- | --- | --- | --- | --- | --- | --- | --- | --- | --- | --- | --- |
|  |  | 4-11 µm | 63-90 µm | pIRIR_290_ | pIRIR_225_ | 4-11 µm | 63-90 µm | pIRIR_290_ | pIRIR_225_ | 4-11 µm | 63-90 µm | 4-11 µm | 63-90 µm | pIRIR_290_ | pIRIR_225_ |
| **BAT 1.0** | 87 | 31 ± 1 (n=19) | 28 ± 1 (n=18) | 80 ± 8 (n = 6) | 63 ± 1 (n = 6) | 1.01 ± 0.01 | 1.0 ± 0.01 | 0.92 ± 0.02 | 1.00 ± 0.01 | 0.97 ± 0.01 | 0.96 ± 0.01 | 0.00 ± 0.29 | 0.15 ± 0.03 | 1.66 ± 0.11 | 2.18 ± 0.09 |
| **BAT 1.1** | 103 | 37 ± 1 (n=12) | 40 ± 2 (n=20) | 87 ± 3 (n = 5) | 71 ± 1 (n = 6) | 1.07 ± 0.06 | 0.98 ± 0.01 | 0.92 ± 0.02 | 1.00 ± 0.02 | 1.01 ± 0.04 | 0.98 ± 0.01 | 0.00 ± 0.25 | 0.17 ± 0.04 | 2.00 ± 0.11 | 2.06 ± 0.12 |
| **BAT 1.7** | 214 | 74 ± 1 (n=15) | 91 ± 6 (n=11) | 133 ± 6 (n = 6) | 117 ± 4 (n = 5) | 0.99 ± 0.02 | 0.99 ± 0.02 | 0.96 ± 0.02 | 1.03 ± 0.02 | 0.97 ± 0.01 | 0.94 ± 0.02 | 0.09 ± 0.04 | 0.10 ± 0.12 | 1.07 ± 0.06 | 1.51 ± 0.12 |
| **BAT 1.8** | 423 | 108 ± 1 (n=13) | 110 ± 6 (n = 14) | 165 ± 4 (n = 6) | 131 ± 9 (n = 5) | 0.95 ± 0.01 | 0.98 ± 0.01 | 0.97 ± 0.02 | 0.96 ± 0.01 | 0.95 ± 0.01 | 0.94 ± 0.01 | 0.10 ± 0.02 | 0.17 ± 0.04 | 1.29 ± 0.05 | 1.28 ± 0.09 |
| **BAT 1.9** | 641 | 145 ± 3 (n=15) | 116 ± 6 (n = 11) | 230 ± 16 (n = 6) | 188 ± 8 (n = 6) | 0.97 ± 0.01 | 0.98 ± 0.01 | 0.94 ± 0.03 | 0.99 ± 0.01 | 0.95 ± 0.01 | 0.95 ± 0.02 | 0.08 ± 0.02 | 0.60 ± 0.28 | 0.77 ± 0.28 | 0.83 ± 0.11 |
| **BAT 1.10** | 821 | 176 ± 2 (n=10) | 168 ± 7 (n = 10) | 261 ± 14 (n = 6) | 215 ± 8 (n = 6) | 0.96 ± 0.01 | 0.96 ± 0.01 | 0.96 ± 0.04 | 0.99 ± 0.01 | 0.97 ± 0.01 | 0.93 ± 0.02 | 0.03 ± 0.01 | 0.02 ± 0.06 | 0.95 ± 0.21 | 0.98 ± 0.08 |
| **BAT 1.11** | 953 | 217 ± 4 (n=13) | 211 ± 8 (n = 12) | 373 ± 12 (n = 11) | 318 ± 22 (n = 6) | 0.96 ± 0.01 | 0.94 ± 0.01 | 0.94 ± 0.02 | 0.98 ± 0.01 | 0.96 ± 0.01 | 0.96 ± 0.02 | 0.06 ± 0.01 | 0.08 ± 0.02 | 1.30 ± 0.12 | 0.77 ± 0.01 |
| **BAT 1.12 A** | 1200 | 237 ± 3 (n=11) | 224 ± 12 (n = 11) | 534 ± 19 (n=10) | 403 ± 18 (n = 5) | 0.94 ± 0.02 | 0.95 ± 0.01 | 0.90 ± 0.01 | 0.94 ± 0.03 | 0.97 ± 0.01 | 0.96 ± 0.01 | 0.04 ± 0.02 | 0.06 ± 0.02 | 0.95 ± 0.06 | 0.92 ± 0.06 |
| **BAT 1.12 B** | 1200 | 206 ± 4 (n=11) | 262 ± 16 (n = 13) | 452 ± 12 (n=10) | 316 ± 11 (n = 6) | 0.95 ± 0.01 | 0.95 ± 0.01 | 0.95 ± 0.01 | 0.97 ± 0.01 | 0.90 ± 0.01 | 0.96 ± 0.01 | 0.05 ± 0.01 | 0.10 ± 0.02 | 0.74 ± 0.04 | 1.05 ± 0.08 |
| **BAT 1.13 A** | 1300 | 275 ± 8 (n=12) | *307 ± 33 (n = 11)* | *787 ± 41 (n=9)* | *598 ± 16 (n = 6)* | 0.99 ± 0.01 | 0.98 ± 0.02 | 0.94 ± 0.02 | 0.98 ± 0.02 | 0.92 ± 0.01 | 0.94 ± 0.02 | 0.07 ± 0.03 | 0.29 ± 0.11 | 0.97 ± 0.02 | 0.94 ± 0.06 |
| **BAT 1.13 B** | 1300 | 345 ± 9 (n=13) | *357 ± 46 (n = 14)* | *789 ± 40 (n = 6)* | *760 ± 33 (n = 6)* | 0.98 ± 0.01 | 0.96 ± 0.01 | 0.89 ± 0.03 | 0.94 ± 0.02 | 0.96 ± 0.01 | 0.97 ± 0.02 | 0.06 ± 0.01 | 0.00 ± 0.02 | 0.84 ± 0.06 | 0.97 ± 0.07 |
| **BAT 1.14 A** | 1450 | 334 ± 7 (n=10) | *280 ± 28 (n = 13)* | *806 ± 63 (n=8)* | *568 ± 16 (n = 6)* | 0.96 ± 0.01 | 1.0 ± 0.02 | 0.90 ± 0.01 | 0.98 ± 0.01 | 0.96 ± 0.01 | 0.92 ± 0.01 | 0.08 ± 0.01 | 0.26 ± 0.12 | 0.98 ± 0.05 | 0.91 ± 0.03 |
| **BAT 1.14 B** | 1450 | 359 ± 11 (n=14) | *291 ± 16 (n = 10)* | *887 ± 68 (n=7)* | *687 ± 41 (n = 6)* | 0.96 ± 0.01 | 0.99 ± 0.02 | 0.88 ± 0.02 | 0.97 ± 0.02 | 0.94 ± 0.01 | 1.01 ± 0.02 | 0.09 ± 0.01 | 0.15 ± 0.15 | 1.06 ± 0.03 | 0.93 ± 0.05 |
| **BAT 1.16** | 1650 | 348 ± 6 (n=10) | *366 ± 53 (n = 10)* | *1181 ± 129 (n=9)* | *716 ± 27 (n = 10)* | 0.95 ± 0.01 | 0.91 ± 0.03 | 0.92 ± 0.02 | 0.98 ± 0.01 | 0.90 ± 0.00 | 0.89 ± 0.01 | 0.07 ± 0.01 | 0.24 ± 0.06 | 0.91 ± 0.02 | 0.74 ± 0.04 |
| **BAT 1.17** | 1900 | 383 ± 12 (n=12) | *333 ± 31 (n = 12)* | *1163 ± 126 (n=6)* | *655 ± 24 (n = 8)* | 0.95 ± 0.01 | 0.94 ± 0.01 | 0.90 ± 0.03 | 0.99 ± 0.02 | 0.96 ± 0.01 | 0.94 ± 0.02 | 0.17 ± 0.06 | 0.66 ± 0.26 | 1.02 ± 0.05 | 0.92 ± 0.07 |
| **BAT 1.18** | 1900 | 386 ± 10 (n=13) | *377 ± 27 (n = 10)* | *1056 ± 107 (n=6)* | *558 ± 51 (n = 6)* | 0.97 ± 0.01 | 0.90 ± 0.02 | 1.0 ± 0.04 | 1.02 ± 0.02 | 0.97 ± 0.01 | 0.93 ± 0.02 | 0.09 ± 0.02 | 0.74 ± 0.11 | 0.89 ± 0.03 | 0.13 ± 0.00 |
| **BAT 1.19 A** | 2400 | 486 ± 5 (n=11) | *345 ± 23 (n= 13)* |  | *1204 ± 66 (n = 8)* | 0.97 ± 0.01 | 0.91 ± 0.01 |  | 0.95 ± 0.02 | 0.95 ± 0.01 | 0.90 ± 0.01 | 0.17 ± 0.05 | 1.57 ± 0.28 |  | 0.80 ± 0.04 |
| **BAT 1.19 B** | 2400 | 475 ± 7 (n=11) | *414 ± 37 (n = 14)* |  | *1270 ± 62 (n = 7)* | 0.97 ± 0.01 | 0.91 ± 0.01 |  | 0.94 ± 0.01 | 0.96 ± 0.01 | 0.91 ± 0.01 | 0.09 ± 0.01 | 0.36 ± 0.05 |  | 0.80 ± 0.03 |
