# Supplementary material for: Testing polymineral post‐IR IRSL and quartz SAR‐OSL protocols on Middle to Late Pleistocene loess at Batajnica, Serbia
Source: Boreas. 2020 May 4;49(3):615–33. doi: 10.1111/bor.12442 (PMC7508060; doi:10.1111/bor.12442)
Supplement: Supplementary file 13 — Table S5. The measured residual doses along with the performance parameters of the pIRIR290 procedure for fine (4–11 μm) polymineral grains of the 10 samples analysed. [file BOR-49-615-s013.docx]

Table S5. The measured residual doses along with the performance parameters of the pIRIR_290_ procedure for fine (4-11 µm) polymineral grains of the ten samples analysed. Three or four aliquots from each sample were exposed to lamp for one month and then the residual dose was measured in the usual manner (same measurement protocol as for equivalent dose determination described in main text Section entitled Luminescence properties – polymineral fine grains). The aliquots with poor recycling ratio (exceeding 10% from unity) were rejected. (*) indicate the equivalent doses measured for the samples with natural signal close to saturation.

| Sample code | Uncorrected De pIRIR_290_ (Gy) | Residual De pIRIR_290_(Gy) | Recycling | Recuperation (%) |
| --- | --- | --- | --- | --- |
| BAT 1.7 | 133±6 (n=6/6) | 5.7±0.5 (n=4/4) | 1.01±0.04 | 9.1±2.0 |
| BAT 1.8 | 165±4 (n=6/6) | 5.6±1.1 (n=4/4) | 1.00±0.02 | 8.2±0.9 |
| BAT 1.9 | 230±16 (n=6/6) | 7.5±1.2 (n=3/4) | 0.96±0.03 | 9.8±1.6 |
| BAT 1.10 | 261±14 (n=6/6) | 10.5±0.7 (n=3/3) | 1.02±0.04 | 5.6±1.6 |
| BAT 1.11 | 373±12 (n=11/11) | 9.1±1.2 (n=2/3) | 1.02±0.07 | 6.1±1.3 |
| BAT 1.12 A | 534±19 (n=10/12) | 8.3±3.2 (n=2/3) | 0.09±0.01 | 2.8±0.2 |
| BAT 1.12 B | 452±12 (n=10/12) | 6.3±0.9 (n=2/3) | 0.92±0.01 | 10.3±1.2 |
| BAT 1.13 A | *787±41 (n=9/11)** | 12.3±0.8 (n=3/3) | 0.90±0.02 | 5.4±1.4 |
| BAT 1.13 B | *789±40 (n=6/6)** | 12.9±1.3 (n=3/3) | 0.96±0.02 | 6.9±1.6 |
| BAT 1.14 A | *806±63 (n=8/9)** | 11.1±1.6 (n=3/3) | 0.91±0.01 | 8.6±0.9 |
