# Supplementary material for: Testing polymineral post‐IR IRSL and quartz SAR‐OSL protocols on Middle to Late Pleistocene loess at Batajnica, Serbia
Source: Boreas. 2020 May 4;49(3):615–33. doi: 10.1111/bor.12442 (PMC7508060; doi:10.1111/bor.12442)
Supplement: Supplementary file 14 — Table S6. The measured residual doses along with the performance parameters of the pIRIR225 procedure for fine (4–11 μm) polymineral grains of the nine samples analysed. [file BOR-49-615-s014.docx]

Table S6. The measured residual doses along with the performance parameters of the pIRIR_225_ procedure for fine (4-11 µm) polymineral grains of the nine samples analyzed. Four or five aliquots from each sample were exposed to lamp for one month and then the residual dose was measured in the usual manner (same measurement protocol as for equivalent dose determination described in main text Section entitled Luminescence properties – polymineral fine grains). (*) indicate the equivalent doses measured for the samples with natural signal close to saturation.

| Sample code | Uncorrected De pIRIR_225_ (Gy) | Residual De pIRIR_225_ (Gy) | Recycling | Recuperation (%) |
| --- | --- | --- | --- | --- |
| BAT 1.10 | 215±8 (n=6/6) | 0.6±0.1 (n=5/5) | 0.76±0.06 | 41±14 |
| BAT 1.11 | 318±22 (n=6/6) | 1.3±0.2 (n=5/5) | 0.91±0.09 | 24±5 |
| BAT 1.12B | 316±11 (n=6/6) | 1.6±0.2 (n=4/4) | 0.75±0.07 | 38±7 |
| BAT 1.14B | *687±41 (n=6/6)** | 1.9±0.4 (n=4/4) | 0.96±0.03 | 28±3 |
| BAT 1.16 | *716±27 (n=10/10)** | 1.8±0.7 (n=4/4) | 0.97±0.02 | 36±4 |
| BAT 1.17 | *655±24 (n=8/9)** | 2.5±0.3 (n=4/4) | 1.01±0.05 | 42±2 |
| BAT 1.18 | *558±51 (n=6/6)** | 2.5±0.4 (n=4/4) | 0.95±0.03 | 36±6 |
| BAT 1.19A | *1204±66 (n=8/8)** | 2.6±0.5 (n=4/4) | 1.02±0.03 | 41±5 |
| BAT 1.19B | *1270±62 (n=7/7)** | 3.2±0.2 (n=4/4) | 0.94±0.02 | 37±2 |
