# Supplementary material for: Testing polymineral post‐IR IRSL and quartz SAR‐OSL protocols on Middle to Late Pleistocene loess at Batajnica, Serbia
Source: Boreas. 2020 May 4;49(3):615–33. doi: 10.1111/bor.12442 (PMC7508060; doi:10.1111/bor.12442)
Supplement: Supplementary file 15 — Table S7. g‐values measured on polymineral fine grains using the pIRIR290 and pIRIR225 protocols as well as on fine and coarse quartz using the SAR‐OSL protocol. [file BOR-49-615-s015.docx]

Table S7. g-values measured on polymineral fine grains using pIRIR_290_ and pIRIR_225_ protocols as well as on fine and coarse quartz using SAR-OSL protocol.

| Sample code | protocol | aliq | g value (%) | Average g-value (%) |
| --- | --- | --- | --- | --- |
| BAT 1.11  4-11 µm polymineral | pIRIR_290_ | 1 | 0.01±1.12 | 1.3±0.6 |
|  |  | 2 | 1.77±1.02 |  |
|  |  | 3 | 2.69±1.08 |  |
|  |  | 4 | 0.64±1.01 |  |
| BAT 1.11  4-11 µm polymineral | pIRIR_225_ | 1 | -0.82±-0.71 | 0.5±0.5 |
|  |  | 2 | 1.14±0.74 |  |
|  |  | 3 | 1.18±0.74 |  |
|  |  | 4 | 0.54±0.76 |  |
| BAT 1.19A  4-11 µm polymineral | pIRIR_225_ | 1 | 2.17±1.59 | 3.2±0.4 |
|  |  | 2 | 2.86±1.71 |  |
|  |  | 3 | 3.48±1.67 |  |
|  |  | 4 | 4.23±1.72 |  |
| BAT 1.11  4-11 µm quartz | SAR-OSL | 1 | 3.11±1.03 | 2.5±0.4 |
|  |  | 2 | 2.71±0.88 |  |
|  |  | 3 | 1.65±1.08 |  |
| BAT 1.11  63-90 µm quartz | SAR-OSL | 1 | 4.28±0.86 | 2.9±0.6 |
|  |  | 2 | 3.24±0.75 |  |
|  |  | 3 | 1.34±0.72 |  |
|  |  | 4 | 2.80±0.08 |  |
