# Supplementary material for: Testing polymineral post‐IR IRSL and quartz SAR‐OSL protocols on Middle to Late Pleistocene loess at Batajnica, Serbia
Source: Boreas. 2020 May 4;49(3):615–33. doi: 10.1111/bor.12442 (PMC7508060; doi:10.1111/bor.12442)
Supplement: Supplementary file 16 — Table S8. The effect of adding large doses on top of the naturally accrued dose for sample BAT‐1.19A using SAR‐OSL, pIRIR225 and pIRIR290 protocols. [file BOR-49-615-s016.docx]

Table S8. The effect of adding large doses on top of the naturally accrued dose for sample BAT-1.19A using SAR-OSL, pIRIR_225_ and pIRIR_290_ protocols. L_n_/T_n_ represents the natural corrected OSL signal. L_n_^*^/T_n_^*^ is the corrected luminescence signal measured after a known dose (3600 Gy) is given on top of the naturally accrued dose, for this old sample. L_x_/T_x_ represents the normalised OSL signal obtained for a large regenerative dose (5000Gy) given after the L_n_^*^/T_n_^*^ was measured. L_x_/T_x_-SAR represents the corrected luminescence signal for a regenerative dose of 5000 Gy measured in the SAR protocol.

| Sample code | Protocol | Added dose | L_n_/T_n_ | (L_n_/T_n_)* | (L_x_/T_x_ SAR) | (L_x_/T_x_) | (L_n_/T_n_) / (L_x_/T_x_ SAR) | (L_n_/T_n_)* / (L_x_/T_x_ ) | (L_n_/T_n_)* / (L_n_/T_n_) |
| --- | --- | --- | --- | --- | --- | --- | --- | --- | --- |
| BAT-1.19A | 4-11 µm quartz SAR-OSL | Nat+4500 Gy | 10.2±0.2 | 19.3±0.3 | 17.3±0.4 | 20.4±0.4 | 0.59±0.02 | 0.95±0.02 | 1.90±0.02 |
|  | 63-90 µm quartz SAR-OSL | Nat+4650 Gy | 5.2±0.2 | 4.9±0.4 | 5.2±0.5 | 4.8±0.3 | 1.00±0.10 | 1.03±0.11 | 0.96±0.08 |
|  | 4-11 µm polymineral pIRIR_225_ | Nat+3600 Gy | 14.4±0.1 | 19.5±0.5 | 17.2±0.1 | 17.7±0.2 | 0.84±0.01 | 1.10±0.03 | 1.35±0.03 |
|  | 4-11 µm polymineral pIRIR_290_ | Nat+3600 Gy | 16.4±0.4 | 19.8±0.8 | 17.2±0.2 | 15.2±0.3 | 1.08±0.03 | 1.15±0.05 | 1.20±0.04 |
