# Supplementary material for: Testing polymineral post‐IR IRSL and quartz SAR‐OSL protocols on Middle to Late Pleistocene loess at Batajnica, Serbia
Source: Boreas. 2020 May 4;49(3):615–33. doi: 10.1111/bor.12442 (PMC7508060; doi:10.1111/bor.12442)
Supplement: Supplementary file 17 — Table S9. Maximum corrected luminescence signals in the SAR‐OSL and pIRIR protocols used and natural corrected luminescence signals for the sample BAT‐1.19A. [file BOR-49-615-s017.docx]

Table S9. Maximum corrected luminescence signals in the SAR-OSL and pIRIR protocols used and natural corrected luminescence signals for the sample BAT-1.19A. The last column presents the ratio of the natural signal to signal obtained for a given dose of 5000Gy, used as an indication of the closeness of the signal to saturation

| Sample code | Grain size | L_n_/T_n_ average | L_x_/T_x5000 Gy_ | (L_n_/T_n_)/(L_x_/T_x 5000Gy_) |
| --- | --- | --- | --- | --- |
| BAT-1.19A | 4-11 µm quartz | 9.6±0.1 n=11 | 17.2±0.4  n=3 | 0.56±0.01 |
|  | 63-90 µm quartz | 5.2±0.2 n=13 | 5.5±0.4^(*)^  n=3 | 0.95±0.08 |
|  | 4-11 µm polymineral pIRIR_290_ | 15.9±0.3 n=9 | 15.2±0.3  n=3 | 1.05±0.03 |
|  | 4-11 µm polymineral pIRIR_225_ | 14.5±0.1 n=8 | 17.2±0.1  n=3 | 0.84±0.01 |

**(*)** The L_x_/T_x_ signal was calculated taking into account both L_x_/T_x_ at 2500Gy and L_x_/T_x_ at 5000 Gy signals.
