# Supplementary material for: Testing polymineral post‐IR IRSL and quartz SAR‐OSL protocols on Middle to Late Pleistocene loess at Batajnica, Serbia
Source: Boreas. 2020 May 4;49(3):615–33. doi: 10.1111/bor.12442 (PMC7508060; doi:10.1111/bor.12442)
Supplement: Supplementary file 18 — Table S10. Saturation characteristics for dose response curves up to 5000 Gy on BAT‐1.19A using different test doses. [file BOR-49-615-s018.docx]

Table S10. Saturation characteristics for dose response curves up to 5000 Gy on BAT-1.19A using different test doses. Data was fitted with a sum of two saturating exponential function.

| Test dose (Gy) | Test dose (% De) | D_01_ (Gy) | D_02_ (Gy) | (L_n_/T_n_)/(L_x_/T_x_) _5000 Gy_ |
| --- | --- | --- | --- | --- |
| 17 | 0.9 | 193 ± 32 | 764 ± 145 | 1.05 ± 0.02 |
| 200 | 11 | 160 ± 34 | 820 ± 83 | 0.95 ± 0.02 |
| 400 | 22 | 257 ± 45 | 1241 ± 277 | 0.92 ± 0.01 |
| 800 | 44 | 232 ± 38 | 1042 ± 135 | 0.89 ± 0.02 |
