# Supplementary material for: Testing polymineral post‐IR IRSL and quartz SAR‐OSL protocols on Middle to Late Pleistocene loess at Batajnica, Serbia
Source: Boreas. 2020 May 4;49(3):615–33. doi: 10.1111/bor.12442 (PMC7508060; doi:10.1111/bor.12442)
Supplement: Supplementary file 19 — Table S11. Natural corrected luminescence signals for the samples BAT‐1.12A–BAT‐1.19B and maximum corrected luminescence signals recorded in the SAR‐OSL and post‐IR IRSL protocols. [file BOR-49-615-s019.docx]

Table S11. Natural corrected luminescence signals for the samples BAT-1.12A-BAT-1.19B and maximum corrected luminescence signals recorded in the SAR-OSL and post-IR IRSL protocols. The last column presents the ratio of the natural signal to signal obtained for the maximum dose given, used as an indication of the closeness of the signal to saturation. * indicates the Ln/Tn average on doublet samples.

| Grain size | Sample code | L_n_/T_n_ average | Maximum given dose (Gy) | L_x_/T_x_ max | (L_n_/T_n_)/(L_x_/T_x 5000Gy_) |
| --- | --- | --- | --- | --- | --- |
| 4-11 µm quartz | BAT 1.12^*^ | 7.01±0.09 | 5000 | 17.64±0.29 | 0.40±0.01 |
|  | BAT 1.13A | 7.57±0.13 | 5000 |  | 0.43±0.01 |
|  | BAT 1.13B | 8.15±0.07 | 5000 |  | 0.46±0.01 |
|  | BAT 1.14A | 7.99±0.10 | 5000 |  | 0.45±0.01 |
|  | BAT 1.14B | 8.50±0.07 | 5000 |  | 0.48±0.01 |
|  | BAT 1.16 | 8.58±0.07 | 5000 |  | 0.49±0.01 |
|  | BAT 1.17^*^ | 8.83±0.06 | 5000 |  | 0.50±0.01 |
|  | BAT 1.19^*^ | 9.74±0.06 | 5000 |  | 0.55±0.01 |
| 63-90 µm quartz | BAT 1.12^*^ | 4.93±0.09 | 2500+500 | 6.49±0.25 | 0.76±0.03 |
|  | BAT 1.13A | 5.67±0.54 | 2500+500 |  | 0.87±0.09 |
|  | BAT 1.13B | 5.57±0.32 | 2500+5000 |  | 0.86±0.06 |
|  | BAT 1.14A | 5.25±0.25 | 2500+5000 |  | 0.81±0.05 |
|  | BAT 1.14B | 5.21±0.31 | 2500+5000 |  | 0.80±0.06 |
|  | BAT 1.16 | 5.20±0.31 | 2500+5000 |  | 0.80±0.06 |
|  | BAT 1.17^*^ | 5.24±0.16 | 2500+5000 |  | 0.81±0.04 |
|  | BAT 1.19^*^ | 5.04±0.16 | 2500+5000 |  | 0.78±0.04 |
| 4-11 µm polymineral pIRIR_290_ | BAT 1.12^*^ | 11.16±0.12 | 5000 | 15.45±0.24 | 0.72±0.01 |
|  | BAT 1.13A | 14.20±0.22 | 5000 |  | 0.92±0.02 |
|  | BAT 1.13B | 14.64±0.19 | 5000 |  | 0.95±0.02 |
|  | BAT 1.14A | 13.85±0.28 | 5000 |  | 0.90±0.02 |
|  | BAT 1.14B | 14.40±0.18 | 5000 |  | 0.93±0.02 |
|  | BAT 1.16 | 14.94±0.26 | 5000 |  | 0.97±0.02 |
|  | BAT 1.17^*^ | 13.87±0.14 | 5000 |  | 0.90±0.02 |
|  | BAT 1.19^*^ | 15.67±0.20 | 5000 |  | 1.01±0.02 |
| 4-11 µm polymineral pIRIR_225_ | BAT 1.12^*^ | 9.66±0.25 | 5000 | 16.01±0.64 | 0.60±0.03 |
|  | BAT 1.13A | 13.66±0.18 | 5000 |  | 0.85±0.04 |
|  | BAT 1.13B | 14.08±0.21 | 5000 |  | 0.88±0.04 |
|  | BAT 1.14A | 12.73±0.12 | 5000 |  | 0.80±0.03 |
|  | BAT 1.14B | 13.96±0.26 | 5000 |  | 0.87±0.04 |
|  | BAT 1.16 | 12.93±0.08 | 5000 |  | 0.81±0.03 |
|  | BAT 1.17^*^ | 11.54±0.25 | 5000 |  | 0.72±0.03 |
|  | BAT 1.19^*^ | 14.24±0.11 | 5000 |  | 0.89±0.04 |
