# Supplementary material for: Testing polymineral post‐IR IRSL and quartz SAR‐OSL protocols on Middle to Late Pleistocene loess at Batajnica, Serbia
Source: Boreas. 2020 May 4;49(3):615–33. doi: 10.1111/bor.12442 (PMC7508060; doi:10.1111/bor.12442)
Supplement: Supplementary file 20 — Data S1. Sample preparation for alpha spectrometry. [file BOR-49-615-s020.docx]

Data S1. Sample preparation for alpha spectrometry

For each 0.5 g of dried homogenized 0.3 ml of ^210^Po (an alpha energy of 4.979 MeV) tracer (with 100 mBq/ml activity) was added in order to determine the chemical yield. Acid digestion was carried out by using 10 ml of concentrated HNO_3_ in three steps; each time the sample evaporated until near-dryness. The same procedure was repeated in the presence of HCl. To eliminate the organic matter, H_2_O_2_ was added. The samples were heated at a constant temperature of 150 ̊ C. In order to eliminate the acids, 20 ml of distilled water was added and evaporated until near-dryness. The procedure was repeated three times. The ^210^Po sources were prepared by spontaneous deposition on the surface of high Ni content stainless steel discs in HCl acid medium with 0.5-1 pH for 3 h at 85 ̊ C. Interferences (Fe-ions) were eliminated by adding ascorbic acid for formatting iron complexes (Begy et al., 2015).

Begy, R.C., Dumitru, O.A., Simon, H., Steopoaie, I., 2015. An improved procedure for the determination of Po-210 by alpha spectrometry in sediments samples from Danube Delta. Journal of Radioanytical and Nuclear Chemistry 303, 2553-2557.
